# A two-step workflow based on plasma p-tau217 to screen for amyloid β positivity with further confirmatory testing only in uncertain cases

**DOI:** 10.1038/s43587-023-00471-5

**Published:** 2023-08-31

**Authors:** Wagner S. Brum, Nicholas C. Cullen, Shorena Janelidze, Nicholas J. Ashton, Eduardo R. Zimmer, Joseph Therriault, Andrea L. Benedet, Nesrine Rahmouni, Cécile Tissot, Jenna Stevenson, Stijn Servaes, Gallen Triana-Baltzer, Hartmuth C. Kolb, Sebastian Palmqvist, Erik Stomrud, Pedro Rosa-Neto, Kaj Blennow, Oskar Hansson

**Affiliations:** 1 Department of Psychiatry and Neurochemistry, Institute of Neuroscience and Physiology, Sahlgrenska Academy at the University of Gothenburg, Mölndal, Sweden; 2 Graduate Program in Biological Sciences: Biochemistry, Universidade Federal do Rio Grande do Sul, Porto Alegre, Brazil; 3 Clinical Memory Research Unit, Department of Clinical Sciences, Malmö, Lund University, Lund, Sweden; 4 Institute of Psychiatry, Psychology and Neuroscience, Maurice Wohl Institute Clinical Neuroscience, King’s College London, London, UK; 5 NIHR Biomedical Research Centre for Mental Health and Biomedical Research Unit for Dementia, South London and Maudsley NHS Foundation, London, UK; 6 Centre for Age-Related Medicine, Stavanger University Hospital, Stavanger, Norway; 7 Department of Pharmacology, Universidade Federal do Rio Grande do Sul, Porto Alegre, Brazil; 8 Graduate Program in Biological Sciences: Pharmacology, Universidade Federal do Rio Grande do Sul, Porto Alegre, Brazil; 9 McGill Centre for Studies in Aging, McGill University, Montreal, Québec Canada; 10 Translational Neuroimaging Laboratory, McGill Research Centre for Studies in Aging, Montreal, Québec Canada; 11 Department of Neurology and Neurosurgery, Faculty of Medicine, McGill University, Montreal, Québec Canada; 12 Neuroscience Biomarkers, Janssen Research & Development, La Jolla, CA USA; 13 Memory Clinic, Skåne University Hospital, Malmö, Sweden; 14 Clinical Neurochemistry Laboratory, Sahlgrenska University Hospital, Mölndal, Sweden

**Keywords:** Alzheimer's disease, Diagnostic markers, Ageing

## Abstract

Cost-effective strategies for identifying amyloid-β (Aβ) positivity in patients with cognitive impairment are urgently needed with recent approvals of anti-Aβ immunotherapies for Alzheimer’s disease (AD). Blood biomarkers can accurately detect AD pathology, but it is unclear whether their incorporation into a full diagnostic workflow can reduce the number of confirmatory cerebrospinal fluid (CSF) or positron emission tomography (PET) tests needed while accurately classifying patients. We evaluated a two-step workflow for determining Aβ-PET status in patients with mild cognitive impairment (MCI) from two independent memory clinic-based cohorts (*n* = 348). A blood-based model including plasma tau protein 217 (p-tau217), age and *APOE* ε4 status was developed in BioFINDER-1 (area under the curve (AUC) = 89.3%) and validated in BioFINDER-2 (AUC = 94.3%). In step 1, the blood-based model was used to stratify the patients into low, intermediate or high risk of Aβ-PET positivity. In step 2, we assumed referral only of intermediate-risk patients to CSF Aβ42/Aβ40 testing, whereas step 1 alone determined Aβ-status for low- and high-risk groups. Depending on whether lenient, moderate or stringent thresholds were used in step 1, the two-step workflow overall accuracy for detecting Aβ-PET status was 88.2%, 90.5% and 92.0%, respectively, while reducing the number of necessary CSF tests by 85.9%, 72.7% and 61.2%, respectively. In secondary analyses, an adapted version of the BioFINDER-1 model led to successful validation of the two-step workflow with a different plasma p-tau217 immunoassay in patients with cognitive impairment from the TRIAD cohort (*n* = 84). In conclusion, using a plasma p-tau217-based model for risk stratification of patients with MCI can substantially reduce the need for confirmatory testing while accurately classifying patients, offering a cost-effective strategy to detect AD in memory clinic settings.

## Abstract (web summary)

Risk stratification based on plasma p-tau217 can substantially reduce the need for invasive or expensive testing when screening for Aβ positivity in patients with cognitive impairment, offering a cost-effective strategy to support an Alzheimer’s disease diagnosis.

## Main

AD is the primary cause of dementia and is neuropathologically defined by the accumulation of extracellular Aβ plaques and intracellular tangles of hyperphosphorylated tau^1–3^. Established AD biomarkers are essential for patient management and will become increasingly important as disease-modifying treatments approach clinical practice^4^. New anti-Aβ therapies have shown promising results in clearing Aβ from the brain^5–7^, leading to approvals of aducanumab and lecanemab by the US Food and Drug Administration (FDA). Confirmation of underlying AD biomarker abnormalities will be key in determining eligibility for disease-modifying treatments in patients with cognitive impairment visiting memory clinics^8^. Nevertheless, the high cost, invasiveness, time-consuming nature and limited availability of CSF and PET biomarkers hamper their widespread use to screen for AD biomarker positivity in memory clinics.

Blood-based biomarkers hold promise to aid in delivering a biomarker-supported AD diagnosis in a minimally invasive and scalable manner^4^. Plasma p-tau species, including p-tau181, p-tau217 and p-tau231, have shown high performance to identify underlying AD^9–11^. Plasma p-tau217 (tau phosphorylated at Thr217) shows the highest fold-changes in Aβ-positive patients with cognitive impairment, thus being less susceptible to analytical variation^10,12–14^. Moreover, plasma p-tau217 is strongly associated with measures of Aβ pathology and its levels change before tau-PET abnormalities are detectable in AD progression^15–17^, making it a feasible candidate to implement as a routine clinical chemistry test to screen for Aβ positivity in memory clinics.

Nevertheless, the implementation of new AD blood biomarkers into a comprehensive diagnostic workflow for detecting Aβ positivity has received less attention, and the Alzheimer’s Association guidelines for appropriate use of AD blood biomarkers recently highlighted the need for objectively evaluating such a strategy^18^. Indeed, even the best-performing blood p-tau biomarkers present a higher group-level overlap than established CSF and PET biomarkers^19,20^. Consequently, handling their results more granularly could potentially reduce the burden of submitting most patients to confirmatory CSF or PET testing. In this context, a model-based approach for interpreting biomarkers alongside clinically relevant information, which is a common strategy in several medical areas^21,22^, might also be well suited when screening for AD^23–25^.

In two independent secondary memory clinic-based cohorts, we evaluated a two-step workflow for detecting brain amyloidosis (as indexed by Aβ-PET) in patients with MCI. Step 1 consisted of a diagnostic model based on plasma p-tau217, age and *APOE* ε4 (apolipoprotein E allele ε4) for risk stratification of Aβ-PET positivity. Step 2 was based on confirmatory testing with CSF Aβ42/Aβ40 only in those patients with uncertain outcomes at step 1. In secondary analyses, this workflow was evaluated using a different plasma p-tau217 immunoassay version in a third cohort, from a distinct geographical setting. We demonstrate that such a two-step workflow can lead to a reduction in the number of confirmatory Aβ tests needed while preserving a high overall accuracy for detecting Aβ-PET status.

## Results

### Participant characteristics

In total, we included 348 MCI participants from BioFINDER-1 (*n* = 136) and BioFINDER-2 (*n* = 212) (Supplementary Table 1). Frequencies of Aβ-PET positivity (BioFINDER-1, 60.3%; BioFINDER-2, 60.8%) and *APOE* ε4 carriership (BioFINDER-1, 49.3%; BioFINDER-2, 55.2%) were similar and both cohorts had fewer females (BioFINDER-1, 35.3%; BioFINDER-2, 42.0%). Included patients from the two cohorts presented similar Mini-Mental State Examination (MMSE) scores, ages and plasma p-tau217 levels (as measured by the Lilly Research Laboratories’ assay unless otherwise specified). Comorbidities were frequent, with frequencies in the combined population (*n* = 348) of 54.0% for cardiovascular disease, 15.8% for diabetes, 37.9% for dyslipidemia and 9.2% for chronic kidney disease (CKD).

### Model development, validation and threshold definition

**Fig. 1:**
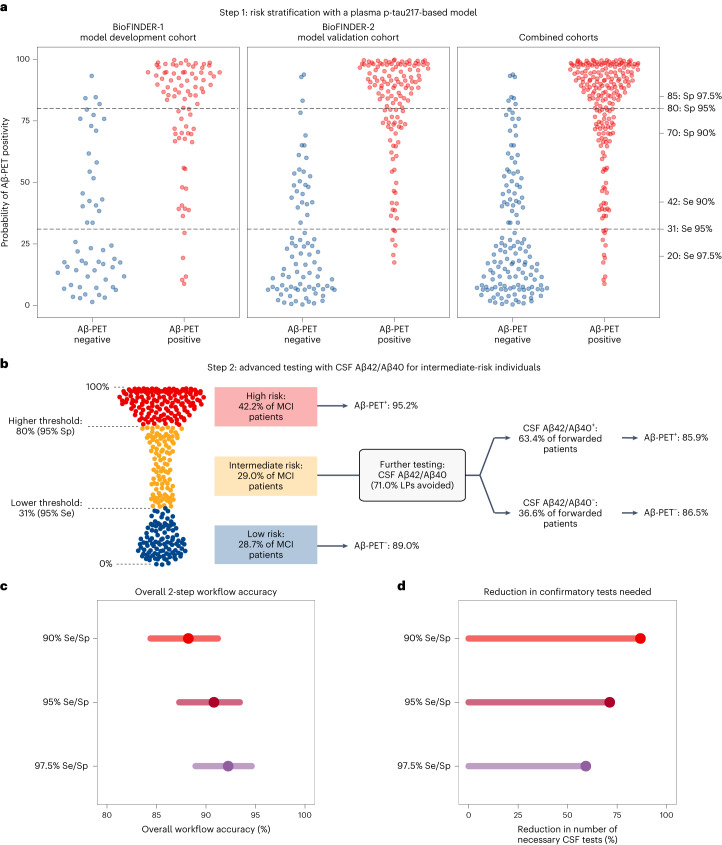
Development and validation of a two-step workflow for Aβ-PET status capable of reducing further confirmatory tests while accurately classifying patients. **a**, Distribution of predicted probabilities of Aβ-PET positivity based on a logistic regression model including plasma p-tau217,age and *APOE* ε4 status as predictors. The predicted probabilities are displayed for the BioFINDER-1 (model training; left), BioFINDER-2 (model validation; middle) and both combined cohorts (right), with blue dots corresponding to individuals who are Aβ-PET negative and red dots to individuals who are Aβ-PET positive. On the right *y* axis, the probability values corresponding to the evaluated risk thresholds are demonstrated and accompanied by the metric used to define them (90%, 95%, 97.5% sensitivity or 90%, 95%, 97.5% specificity). The lower dashed line demonstrates where the 95% sensitivity low-risk threshold falls on the probability distribution, with the upper line corresponding to the 95% specificity high-risk threshold. **b**, Flowchart recapitulating results from the first step of the workflow (blood-biomarker-based risk stratification) and demonstrating the accuracy for the second step of the clinical workflow, when intermediate-risk individuals are referred to lumbar puncture (LP) to perform a CSF Aβ42/Aβ40 test for predicting Aβ-PET status based on the 95% Se/Sp strategy, with the flowchart for the two other strategies presented in Supplementary Information. **c**, The accuracy of both workflow steps combined, corresponding to the proportion of correct classifications for the low- and high-risk groups, along with the proportion of correct CSF Aβ42/Aβ40 classifications in the intermediate-risk group, according to each of the strategies, computed in the BioFINDER-1 and BioFINDER-2 MCI combined populations (*n* = 348). The error bars correspond to 95% CIs. **d**, Dots and lines indicating the observed percentage of reduction in further tests (here using CSF Aβ42/Aβ40) by applying the blood-based risk stratification strategy, based on each of the risk threshold strategies (90% Se/Sp, *n* = 301; 95% Se/Sp, *n* = 247; 97.5% Se/Sp, *n* = 205).

**Extended Data Fig. 1:**
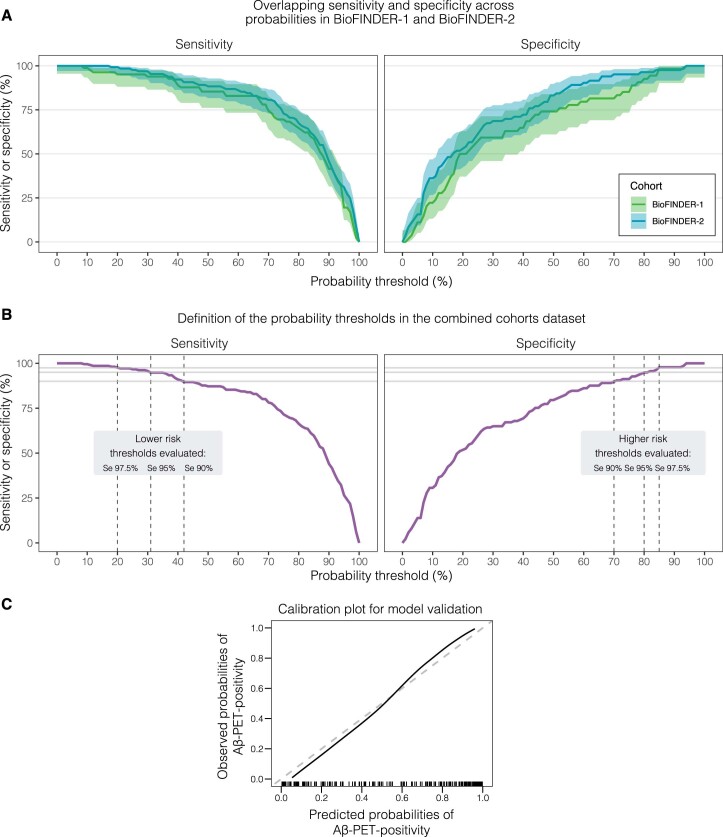
Sensitivity and specificity across possible probability thresholds in both cohorts and derivation of probability thresholds. **(a)** Sensitivity and specificity across probability thresholds in BioFINDER-1 and BioFINDER-2, separately presented. The x-axis corresponds to the full range of possible thresholds for the probabilities of Aβ-PET positivity based on a plasma p-tau217-based model for Aβ-PET positivity. Solid lines correspond to the observed sensitivities and specificities point estimates for the range of possible probability thresholds, and ribbons to 95% confidence intervals, with BioFINDER-1 in light green and BioFINDER-2 in dark green. **(b)** Since sensitivity and specificity overlapped across the range of possible thresholds in both cohorts, we derived risk stratification thresholds based on predictions from both datasets combined. The lower-risk probability thresholds (left) evaluated were 42% (resulting in a sensitivity of 90%), 31% (resulting in a sensitivity of 95%) and 20% (resulting in a sensitivity of 97.5%), while the higher-risk probability thresholds (right) evaluated were 70% (resulting in a specificity of 90%), 80% (resulting in a specificity of 95%) and 85% (resulting in a specificity of 97.5%). **(c)** Calibration plot showing external validation in BioFINDER-2 of the model derived in BioFINDER-1. The solid black line shows smoothed associations between the predicted probabilities and observed frequencies of Aβ-PET-positivity. The closer this line is to the dotted grey identity line, the better performing and more generalizable a prediction model is. Aβ = Amyloid-β. PET = Positron emission tomography. P-tau217 = tau phosphorylated at threonine 217. Se = Sensitivity. Sp = Specificity.

Plasma p-tau217, age and *APOE* ε4 status were evaluated as candidate predictors for developing a logistic regression model for Aβ-PET positivity with bootstrapped backward variable elimination in BioFINDER-1 (Supplementary Table 2). The full model, including plasma p-tau217, age and *APOE* ε4, was selected, presenting an optimism-corrected AUC of 89.3% (95% confidence interval (CI) = 83.7–93.8%) for Aβ-PET positivity in BioFINDER-1. At external validation in BioFINDER-2, an independent cohort, the model also presented high discriminatory performance (AUC = 94.3%, 95% CI = 91.2–97.4%). Next, three different thresholding strategies were explored to classify participants into groups with low, intermediate and high risk of Aβ-PET positivity, based on the plasma p-tau217 model-derived probabilities of Aβ-PET positivity. We defined lower probability thresholds with 90%, 95% and 97.5% sensitivity (to avoid missing detection of patients who are Aβ positive), and higher probability thresholds with 90%, 95% and 97.5% specificity (to avoid classifying patients who are Aβ negative as ‘high risk’). As the model validated well and displayed good calibration, probability thresholds were derived for the combined BioFINDER-1 and BioFINDER-2 dataset (*n* = 348) (Extended Data Fig. 1). Predicted probabilities of Aβ-PET positivity and the resulting thresholds are shown in Fig. 1a.

### Step 1: risk stratification with the blood-based model

Next, we assessed the performance of such thresholds according to the Aβ-PET status (Table 1). We evaluated the accuracy of the low-risk thresholds (specifically, the negative predictive values) by determining the percentage of individuals who are Aβ-PET negative falling below the three different low-risk thresholds. For the more lenient (sensitivity (Se), 90%), intermediate-stringency (Se, 95%) and most stringent (Se, 97.5%) low-risk thresholds evaluated, the accuracy for Aβ-PET negativity was, respectively, 82.0% (18% false negatives), 89.0% (11.0% false negatives) and 93.4% (6.6% false negatives). The accuracy for Aβ-PET positivity (the positive predictive values) of the high-risk thresholds was evaluated by determining the percentage of individuals who are Aβ-PET positive above the different high-risk thresholds. For the more lenient (specificity (Sp), 90%), intermediate-stringency (Sp, 95%) and most stringent (Sp, 97.5%) high-risk thresholds evaluated, the accuracy for Aβ-PET positivity was, respectively, 92.2% (7.8% false positives), 95.2% (4.8% false positives) and 97.7% (2.3% false positives).

**Table 1:** Model-based risk stratification for Aβ-PET positivity according to the three threshold strategies evaluated

	Participants in each risk group (*n*)	Within-risk group Aβ-PET status	
Risk groups		Aβ-PET negative (*n* (%))	Aβ-PET positive (*n* (%))
**90% Se lower-risk threshold** **(%)** **90% Sp higher-risk threshold (%)**			
Low risk (<42)	122	100 (82.0)	22 (18.0)
Intermediate risk (42–70)	47	23 (48.9)	24 (51.1)
High risk (>70)	179	14 (7.8)	165 (92.2)
**95% Se lower-risk threshold** **(%)** **95% Sp higher-risk threshold (%)**			
Low risk (<31)	100	89 (89.0)	11 (11.0)
Intermediate risk (31–80)	101	41 (40.6)	60 (59.4)
High risk (>80)	147	7 (4.8)	140 (95.2)
**97.5% Se lower-risk threshold** **(%)** **97.5% Sp higher-risk threshold (%)**			
Low risk (<20)	76	71 (93.4)	5 (6.6)
Intermediate risk (20–85)	143	63 (44.1)	80 (55.9)
High risk (>85)	129	3 (2.3)	126 (97.7)

Data are presented as *n* or *n* (%). The first column indicates each of the evaluated strategies for blood-biomarker-based risk stratification, with each strategy’s probability threshold indicated in parenthesis next to the low-, intermediate- and high-risk groups. For each of the thresholding strategies, the second column corresponds to the number of screened individuals falling in each risk category. Lastly, Aβ status is shown for low-, intermediate- and high-risk groups. The percentage of Aβ negatives in the low-risk group and the percentage of Aβ positives in the high-risk group correspond to each evaluated threshold’s NPV and PPV, respectively.

When performing risk stratification, the same sensitivity and specificity thresholds were always tested together (for example, 90% Se with 90% Sp, referred to as Se/Sp 90%). As expected, the size of the intermediate-risk group increased when more stringent screening strategies were used: with the more lenient strategy of paired Se/Sp 90% thresholds, 13.5% (*n* = 47 out of 348) of individuals were classified as intermediate risk using the blood-based model; with the Se/Sp 95% thresholding strategy, 29.0% of individuals with MCI (*n* = 101 out of 348) fell into the intermediate-risk group; and with the most stringent strategy of Se/Sp 97.5% thresholds, a larger proportion of individuals, 41.1% (*n* = 143 out of 348), was classified as intermediate risk. For each strategy, the summed percentage of individuals classified into the low- or high-risk groups corresponds to the proportion of patients not needing a confirmatory CSF test, discussed in detail below, alongside overall workflow accuracy.

### Step 2: effect of CSF tests for the intermediate-risk group

**Extended Data Fig. 2:**
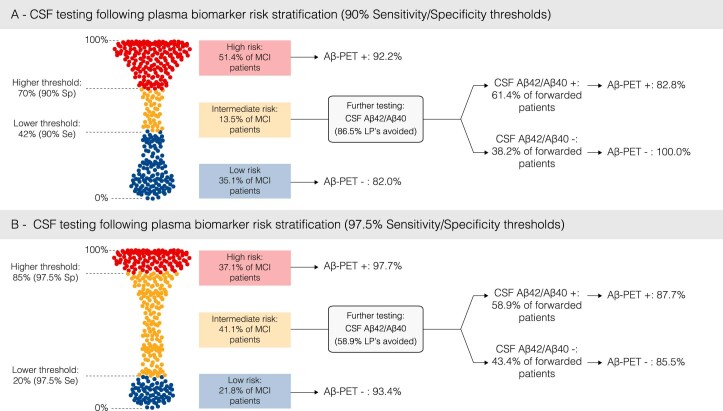
Flowchart illustrating plasma-based risk stratification and further testing of intermediate-risk individuals with CSF Aβ42/Aβ40 for the 90 and 97.5% strategies. Flowchart recapitulating results from the first step of the workflow (blood biomarker-based risk stratification) and demonstrating the accuracy for the second step of the clinical workflow, when intermediate-risk individuals are referred to CSF Aβ42/Aβ40 test for predicting Aβ-PET status. **(a)** Shows results for the 90% Se/Sp risk stratification strategy, and **(b)** for the 97.5% Se/Sp strategy. The 95% Se/Sp strategy is represented in Fig. 1b of the main text. Aβ = Amyloid-β. PET = Positron emission tomography. CSF = cerebrospinal fluid. P-tau217 = tau phosphorylated at threonine 217. Se = Sensitivity. Sp = Specificity. LP = lumbar puncture.

Considering that the patients classified as intermediate risk at the blood-based risk stratification step were patients with uncertain blood-biomarker outcomes, where Aβ-PET positivity ranged from 51% to 59%, we investigated whether fully automated CSF Aβ42/Aβ40 tests would accurately determine the Aβ-PET status in this subgroup. This approach led to a high concordance between a CSF Aβ42/Aβ40 and Aβ-PET status in this group of patients. For the 13.5% of patients with MCI in the intermediate-risk group when using the Se/Sp 90% strategy of the blood-based model, a positive CSF Aβ42/Aβ40 test had a positive predictive value (PPV) of 82.8% for Aβ-PET positivity, whereas a negative CSF Aβ42/Aβ40 test had a negative predictive value (NPV) of 100.0% for Aβ-PET negativity (Extended Data Fig. 2a). For the Se/Sp 95% blood-based stratification strategy, 29.0% of patients with MCI fell into the intermediate-risk group and CSF Aβ42/Aβ40 showed a PPV of 85.9% for Aβ-PET positivity and an 86.5 NPV for Aβ-PET negativity (Fig. 1b). For the 41.1% of patients with MCI classified as intermediate risk with the Se/Sp 97.5% strategy, CSF Aβ42/Aβ40 showed a PPV of 87.7% for Aβ-PET positivity and an 85.5% NPV for Aβ-PET negativity (Extended Data Fig. 2b). In a sensitivity analysis comparing alternative CSF biomarkers to determine Aβ-PET status in this uncertain group, Aβ42/Aβ40 remained as the biomarker with the highest overall accuracy compared with Aβ42 alone or p-tau181/Aβ42 (Supplementary Table 3).

### Workflow overall accuracy and reduction in necessary CSF tests

**Extended Data Fig. 3:**
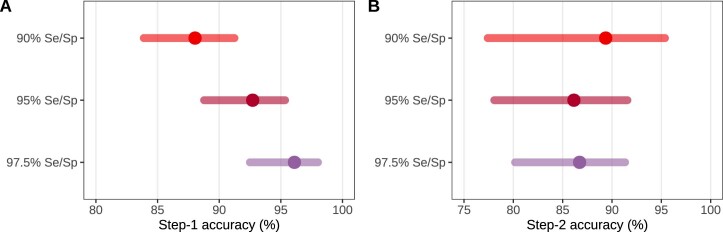
Separate-step performance. For each of the graphics, the x-axis corresponds to the three evaluated strategies for blood-based biomarker risk stratification (Se/Sp 90%; Se/Sp 95%; Se/Sp 97.5%), with dots representing point estimates and bars corresponding to 95% confidence intervals, computed for the BioFINDER-1 and BioFINDER-2 combined population (n = 348). **(a)** Indicates the overall accuracy for the low- and high-risk groups for the workflow’s first step, that is blood-based biomarker risk stratification. This metric was calculated based on the number of Aβ-PET negative individuals classified to the low-risk group and of Aβ-PET positive individuals classified to the high-risk group (90% Se/Sp: n = 265; 95% Se/Sp: n = 229; 97.5% Se/Sp: n = 197), divided by the total individuals in the high and low-risk groups. **(b)** Displays the accuracy for the second step of the workflow. Individuals in the intermediate-risk group were assumed to be forwarded to a lumbar puncture to test CSF Aβ42/40 test, and the accuracy corresponding to the overall concordance of a CSF-negative result with a negative Aβ-PET scan and of a CSF-positive results with a positive Aβ-PET scan (90% Se/Sp: n = 42; 95% Se/Sp: n = 87; 97.5% Se/Sp: n = 143). Aβ = Amyloid-β. PET = Positron emission tomography. CSF = cerebrospinal fluid. P-tau217 = tau phosphorylated at threonine 217. Se = Sensitivity. Sp = Specificity.

In general, more stringent screening strategies led to a higher workflow accuracy (Fig. 1c), but also increased the size of the intermediate-risk group who needed further testing (Fig. 1d). When applying the more lenient screening strategy (Se/Sp 90%), the total proportion of correct Aβ-PET status classifications achieved by the whole two-step workflow (that is, correct blood-based classifications for low- and high-risk groups plus correct CSF Aβ42/Aβ40 classifications for the intermediate-risk group) was 88.2% (95% CI = 84.4–91.2%). Furthermore, this approach reduced the number of patients needed to be referred for a lumbar puncture by 85.9%. With the Se/Sp 95% risk stratification strategy, the overall accuracy of the two-step workflow increased to 90.5% (95% CI = 87.3–93.4%), while reducing the number of patients who needed confirmatory CSF testing by 72.7%. The more stringent screening strategy (Se/Sp 97.5%) presented the highest overall workflow accuracy of 92.7% (95% CI = 88.9–94.6%), while still reducing the number of patients who needed to be referred to confirmatory testing by 61.2%. Accuracies for each of the workflow steps are presented separately in Extended Data Fig. 3.

### Interassay and geographical validation of the workflow

**Fig. 2:**
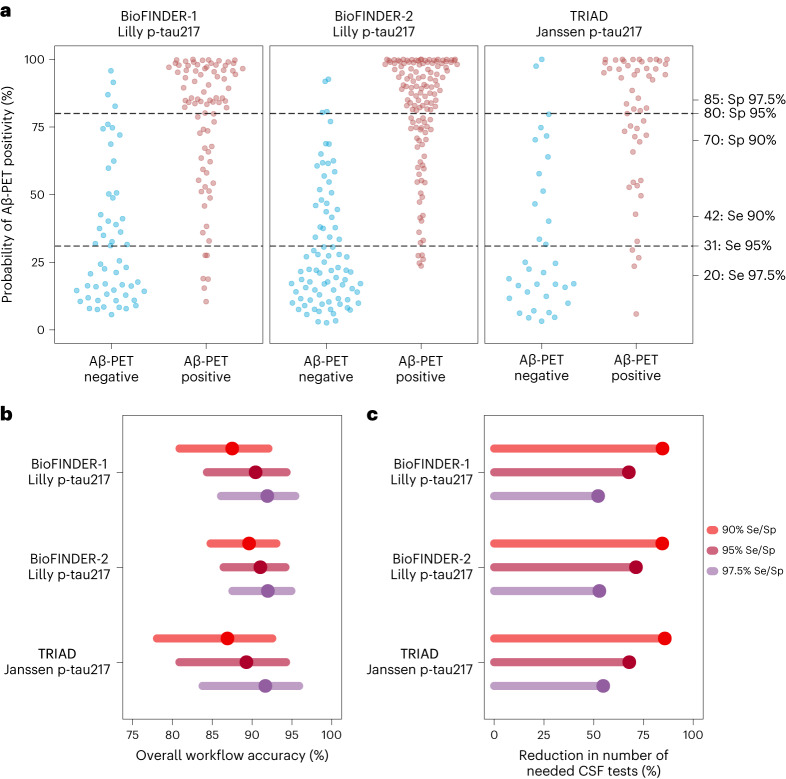
A model with *z*-transformed plasma p-tau217 levels enables interassay and geographical application of the two-step workflow. **a**, Distribution of predicted probabilities of Aβ-PET positivity based on a logistic regression model including *z*-transformed plasma p-tau217 levels, combined with age and *APOE* ε4. The *z*-transformation was done using a CU reference sample from each specific cohort, based on the mean and s.d. of each specific assay in its corresponding population. The predicted probabilities are displayed for the BioFINDER-1 (model training; left), BioFINDER-2 (model validation; middle) and TRIAD (geographical and interassay validation; right), with blue dots corresponding to individuals who are Aβ-PET negative and red dots to individuals who are Aβ-PET positive. On the right *y* axis, the probability values corresponding to the evaluated risk thresholds are demonstrated and accompanied by the metric used to define them (90%, 95%, 97.5% sensitivity or 90%, 95%, 97.5% specificity), and the original thresholds from main analyses were used to evaluate their robustness. The lower dashed line demonstrates where the 95% sensitivity low-risk threshold falls on the probability distribution, with the upper line corresponding to the 95% specificity high-risk threshold. **b**, The accuracy of both workflow steps combined, corresponding to the proportion of correct classifications for the low- and high-risk groups along with the proportion of correct CSF Aβ42/Aβ40 classifications in the intermediate-risk group. The dots correspond to the point estimates for observed accuracy and the lines to 95% CIs, computed based on each cohort’s full sample (BioFINDER-1, *n* = 136; BioFINDER-2, *n* = 212; TRIAD, *n* = 84). Each of the threshold strategies is represented by a color as indicated on the right. **c**, The percentage of reduction in further tests by applying the blood-based risk stratification strategy, based on each of the risk threshold strategies. The dots and lines correspond to the observed reduction in needed confirmatory tests (cohort (number of tests avoided according to 90%, 95% and 97.5% strategies, respectively): BioFINDER-1 (115, 92, 71); BioFINDER-2 (179, 151, 112); and TRIAD (72, 57, 46)).

Finally, we re-fitted the original BioFINDER-1 model but replaced plasma p-tau217 concentrations with plasma p-tau217 values *z*-transformed, based on reference, cognitively unimpaired (CU), Aβ-negative populations, to enable interassay validation (model details in Supplementary Table 4), with successful interassay and geographical validation (Fig. 2 and Supplementary Tables 5 and 6). In both BioFINDER-1 and BioFINDER-2, use of *z*-transformed values of plasma p-tau217 showed similar figures to those of the original concentration-based model, with the following results reported for the 95% Se/Sp strategy with the same thresholds from previous analyses. In BioFINDER-1, the workflow based on the *z*-scored model showed an accuracy of 90.4% (95% CI = 84.3–94.3%) for Aβ status while reducing further testing by 67.6%. Similarly, when applying this model in BioFINDER-2, the workflow reached an overall accuracy of 91.0% (95% CI = 86.4–94.2%), while reducing the number of necessary confirmatory CSF tests by 71.2%. Furthermore, we used this adapted BioFINDER-1 model to obtain risk probabilities in a sample of patients with cognitive impairment (*n* = 84) from the Translational Biomarkers in Aging and Dementia (TRIAD) cohort (McGill University, Canada) with complete biomarker availability and plasma p-tau217 measured with another immunoassay version (Janssen R&D), *z*-transformed based on an internal reference sample of CU Aβ negatives in TRIAD (demographic characteristics in Supplementary Table 7). When applying the model trained in BioFINDER-1 in TRIAD, using the original BioFINDER 95% Se/Sp thresholds, a similarly high overall workflow accuracy was achieved (89.3%, 95% CI = 80.9–94.3%) while reducing the number of necessary confirmatory tests by 67.9%.

## Discussion

**Fig. 3:**
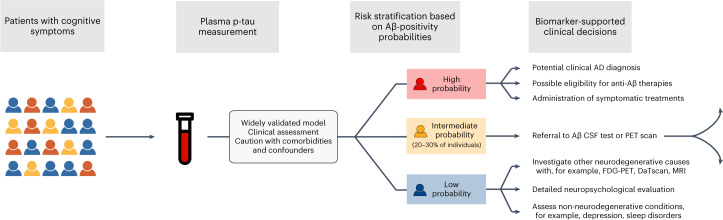
A potential workflow for incorporating a plasma p-tau217 risk prediction model for predicting Aβ status in clinical practice. Conceptual flowchart for future implementation of the proposed two-step diagnostic workflow. Participants with cognitive impairment in specialized settings could be screened for the risk of underlying Aβ pathology based on a high-performance plasma p-tau biomarker model also incorporating clinically relevant variables, such as age and *APOE* *ε*4 status. Importantly, a clinical assessment would determine the need for an AD biomarker assessment. Comorbidities potentially affecting circulating biomarker levels should also be taken into consideration. Based on probability thresholds, chosen according to the decision to be made by the physician, patients could be stratified into low, intermediate and high risk of harboring underlying cerebral Aβ pathology. This biomarker-supported risk stratification could enable highly accurate decisions for individuals in the low- and high-risk groups. Individuals falling within the intermediate-risk group should be forwarded for further testing to determine their Aβ status with a confirmatory PET or CSF Aβ test, depending on center preference and availability. Such a strategy would largely reduce the number of further tests needed, while maintaining a high classification accuracy.

In the present study, we evaluated an efficient two-step diagnostic workflow for the identification of brain Aβ-PET status in patients with MCI using risk stratification based on a blood-biomarker model containing plasma p-tau217, age and *APOE* ε4 status (step 1), followed by confirmatory testing with CSF Aβ42/Aβ40 only in patients with intermediate risk at the first blood-based screening step (step 2). In step 1, risk stratification for Aβ-PET positivity was done based on strategies with varying stringency, leading to accurate classifications for Aβ negativity within the low-risk group and for Aβ-positivity in the high-risk group. This was achieved while keeping the intermediate-risk (‘uncertain’) group reasonably small, substantially reducing the need for further confirmatory testing (reductions from 61.2% to 85.9%). These results indicate that this workflow might substantially reduce the number of patients who need advanced testing using CSF biomarkers or PET scans, while maintaining a high overall classification accuracy (88.2–92.0%). Furthermore, the two-step workflow showed a similarly high performance when using a different p-tau217 immunoassay in TRIAD, in a different geographical setting. A conceptual flowchart for the future application of the proposed two-step workflow is presented in Fig. 3.

Through this two-step workflow, we propose that one way to implement biomarkers in memory clinics could be by using blood biomarkers in risk-prediction models as a first-line screening tool for patients with memory complaints, provided the clinical presentation warrants an AD-specific blood test. The results achieved with our proposal are in line with the recent Alzheimer’s Association guidelines on the appropriate use of blood biomarkers, which stated that one of the challenges of the field was to evaluate whether blood-based assessments for AD pathology could achieve high accuracy (90–95%) so that only uncertain cases would be referred for confirmatory CSF or PET tests^18^. Although the blood-based model and thresholds herein evaluated are not intended to be the final ones to be used in clinical practice, the evaluated strategies provide a practical example that more rigorous screening thresholds lead to higher accuracy, but simultaneously require advanced testing to be done in more patients. Considering the high accuracy both to rule in and to rule out AD observed in step 1 with these example threshold strategies, we assumed that blood-biomarker-supported decisions could be made for participants in the low- and high-risk groups.

Clinical decisions for the participants within the low-risk group could vary. Depending on clinical manifestations, patients could come back to the memory clinic in 6–24 months for another assessment and blood draw. Alternatively, patients and caregivers could be reassured that AD is unlikely to be the cause of the symptoms and investigation of whether the patient has another neurodegenerative disease would be warranted. For instance, an [^18^F]fluorodeoxyglucose (FDG) PET scan could be appropriate for patients with a suspected frontotemporal dementia disorder, a dopamine transporter scan (DaTscan) for those with possible Lewy body etiology and magnetic resonance imaging (MRI) for patients with suspected vascular dementia. In cases where a non-neurodegenerative cause is suspected, a detailed investigation could include further neuropsychological testing and should focus on other possible (and sometimes reversible) causes of worsening in cognitive function, such as depression, post-traumatic stress disorder, substance abuse, delirium, sleep apnea and so on (Fig. 3).

High-risk participants who are very likely to have AD because the etiology causing the symptoms could be clinically diagnosed with greater confidence, allowing for quicker initiation of available treatments than if CSF or PET testing was required. This applies to current symptomatic treatments and, potentially, to new disease-modifying therapies. Even when anti-Aβ therapies obtain coverage by health systems globally, Aβ-PET might not always be a clinical option given the high costs and limited availability. Thus, determining the feasibility of delivering new therapies solely based on blood biomarkers and related algorithms is needed. Ongoing trials, such as TRAILBLAZER-3 (NCT05026866), enrolling participants only with plasma p-tau217, will further aid in clarifying whether anti-Aβ immunotherapies can potentially be delivered without advanced testing. It is important to note that using plasma p-tau217 in a screening diagnostic model alongside other predictors does not exclude the need for interpreting biomarker concentration results alone, because they closely reflect dynamic brain pathological changes, and evaluation of their concentrations alone could also be useful to clinically monitor disease progression and treatment response in the future^26,27^.

In the second step of the workflow, we evaluated CSF Aβ42/Aβ40 as a confirmatory diagnostic test of Aβ-PET status in patients with uncertain (intermediate-risk), blood-biomarker-based outcomes. On widespread implementation of such a workflow, the choice confirmatory test will depend on patient and physician preferences, as well as center availability. CSF testing has the advantage of being simpler and more widely available in secondary memory clinics due to its low infrastructural complexity, in comparison to imaging procedures. In centers where lumbar punctures are not usually performed and a PET scan is not a possible referral, patients could be referred to a tertiary clinic for a lumbar puncture. Costs for Aβ-PET might still be a complicating factor, because it is still mostly used in research and healthcare system coverage is still limited for clinical purposes, as in the USA^28^, whereas CSF tests are covered and widely used in European countries, for instance^29^.

Plasma p-tau217 was chosen as the main blood-biomarker predictor in the screening model for Aβ positivity for being a robust AD-specific biomarker with a large fold-change in Aβ positive patients with cognitive impairment^10^, consistently outperforming other p-tau markers in comparison studies^12,13,30^. As tangle accumulation is more associated with cognitive worsening in the symptomatic phases of AD, another advantage of p-tau217 is that it seems to be driven by both Aβ and tau pathologies^31^. Other blood biomarkers such as p-tau231 and Aβ42/Aβ40 seem to plateau with early Aβ accumulation, besides potential robustness issues due to the very limited AD-related fold-change (around an 8–14% reduction) for the latter^32,33^, compared with fold-changes usually >200% for different plasma p-tau217 assays^10,12,13^. Although it is not yet determined which plasma p-tau217 assays will be implemented on a large scale, we demonstrated the workflow’s performance to be robust using two different, validated, p-tau217 immunoassays^12,13,34^. This shows that such a model could potentially be used based on the locally available plasma p-tau217 assay, with biomarker levels *z*-transformed based on each center’s cognitively unimpaired Aβ-negative reference sample. Both immunoassays demonstrated comparable performance across cohorts (with wider CIs in TRIAD due to lower sample size), although specific assay comparisons were not within the scope of the present work. Importantly, the probability thresholds derived in the concentration-based model performed well between assays without the need for re-optimization, with the workflow demonstrating similar performance both within two independent cohorts from the same geographical setting (BioFINDER-1 and BioFINDER-2) and in a memory clinic-based cohort from a different continent (TRIAD).

**Extended Data Fig. 4:**
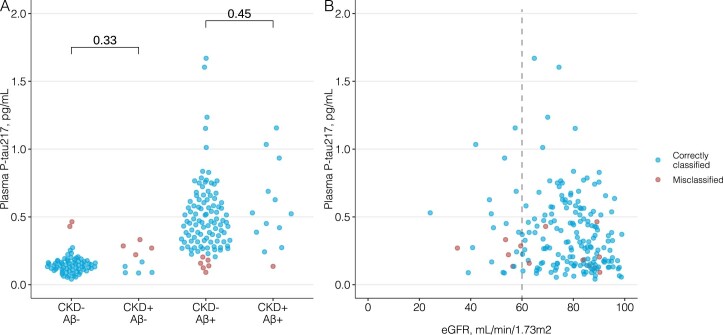
Reduced renal function does not seem to influence plasma p-tau217 levels between Aβ-PET negative and positive individuals classified as low- or high-risk. **(a)** The dots represent plasma p-tau217 concentrations (y-axis), with the x-axis representing the Aβ-PET status in combination with chronic kidney disease (CKD) as determined by an eGFR below 60 mL/min/1.73m2¬. Only participants classified as low- or high-risk at step-1 of the workflow based on the plasma p-tau217 model-derived probabilities and the 95% Se/Sp strategy are included. The colors indicate whether patients were correctly classified (blue) or misclassified (red). P-values come from t-tests (two-sided, alpha 0.05) were used to assess whether plasma p-tau217 levels were altered by the presence of CKD among Aβ-negatives and Aβ-positive participants. Plasma p-tau217 levels did not were not significantly altered by CKD among Aβ-negative participants or among Aβ-positive participants. **(b)** The y-axis represents plasma p-tau217 levels and the x-axis represents continuous values of eGFR, with the with the colors indicating whether patients were correctly classified (blue) or misclassified (red) at the step-1 of the workflow based on the plasma p-tau217 model-derived probabilities and the 95% Se/Sp strategy. The plot shows that misclassifications occur throughout all the span of renal function. Further, the dashed line indicates most of the misclassified individuals with CKD were, in fact, very close to the eGFR cutoff for CKD of 60 mL/min/1.73m2¬. Aβ = Amyloid-β. PET = Positron emission tomography. eGFR = estimated glomerular filtration rate. CKD = chronic kidney disease. P-tau217 = tau phosphorylated at threonine 217. Se = Sensitivity. Sp = Specificity.

**Extended Data Fig. 5:**
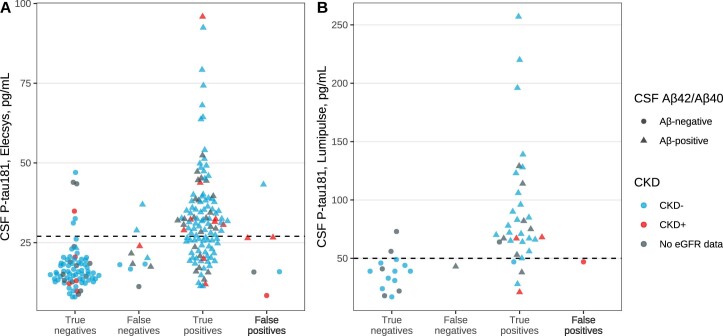
Patients with CKD misclassified as false-positives often presented high CSF p-tau levels or CSF-positivity for Aβ42/Aβ40. This figure represents CSF p-tau181 levels and CSF Aβ42/Aβ40 status measured for BioFINDER-1 and BioFINDER-2 patients according to their classification status at the 95% Se/Sp risk stratification strategy with the main analysis plasma p-tau217-based model. The y-axis and dots displays CSF p-tau181, with colors representing CKD status (CKD-, blue; CKD + , red) and the shapes correspond to CSF Aβ42/Aβ40 status. In the x-axis, patients are stratified into true-negatives (low-risk label at step-1 who were also Aβ-PET-negative), false-negatives (low-risk label at step-1 who were Aβ-PET-positive), true-positives (high-risk label at step-1 who were also Aβ-PET-positive), false-positives (high-risk label at step-1 who were Aβ-PET-negative), with intermediate-risk individuals excluded from the plot (assumed to be referred for a CSF test with no applicable correct/incorrect classification label). **(a)** Displays CSF biomarker results measured with Elecsys for BioFINDER-1 and most of BioFINDER-2 patients, with the horizontal line corresponding to a previously validated cut-off for p-tau181 of 28 pg/mL (ref. ^56^). **(b)** Displays CSF biomarker results measured with Lumipulse a subset of BioFINDER-2 patients, with the horizontal line corresponding to a previously validated cut-off for p-tau181 of 50.2 pg/mL (ref. ^59^). For both assays, CSF Aβ42/Aβ40 was handled as described in the methods. Given the false-positive group (n = 7; x-axis, in bold) had demonstrated a higher rate of CKD after classification with a plasma p-tau217 risk stratification model, this figure indicates that n = 3 out of the n = 4 false-positives with CKD had elevated CSF p-tau181 levels (very close to indicated clinical cutoffs), with n = 2 of these patients also being positive for CSF Aβ42/Aβ40. This suggests a peripheral increase in plasma p-tau217 in the absence of Aβ-PET-positivity could be related to an underlying disease process (since CSF changes might occur earlier than PET) rather than peripheral impaired clearance. Aβ = Amyloid-β. CSF = cerebrospinal fluid. PET = Positron emission tomography. CKD = chronic kidney disease. P-tau181 = tau phosphorylated at threonine 181. P-tau217 = tau phosphorylated at threonine 217. Se = Sensitivity. Sp = Specificity.

Previous reports indicate that, although with varying effect sizes, CKD might be positively associated with plasma p-tau levels^35–37^. Indeed, we found a higher frequency of CKD in the false-positive group with the 95% Se/Sp strategy with the plasma p-tau217 (Lilly) original model (Supplementary Tables 8 and 9). However, misclassifications were not frequent and generally occurred throughout the whole span of renal function, with most of the misclassified patients with CKD being, in fact, close to the estimated glomerular filtration rate cutoff for abnormal renal function (Extended Data Fig. 4). Furthermore, these false-positive patients with CKD often showed up as CSF positive for Aβ42/Aβ40 with elevated CSF p-tau levels (Extended Data Fig. 5), possibly suggesting an early disease process rather than a peripheral confounding effect. Although these and previous results may nevertheless recommend some caution when interpreting plasma p-tau in patients with comorbidities, it seems difficult to determine whether reduced renal function might have truly impacted false positivity in our study in light of the above-mentioned patient-level information.

Traditionally, CSF and PET diagnostic biomarkers for AD have been interpreted by clinicians as binary results (normal/abnormal) and they have not been largely used for risk stratification with prediction models. Despite being excellent proxies of AD pathology, new p-tau blood biomarkers do not present a clear bimodal distribution between non-AD and AD groups and, importantly, they present higher group-level overlaps than CSF and PET Aβ biomarkers^10,38,19^. In consequence, searching for an ‘optimal’ binary cutoff for blood biomarkers might be difficult. In this context, inclusion of other easily accessible variables could help to mitigate the group-overlap issue, and use of different cutoffs with a specific clinical goal (for example, screen-out or screen-in AD) might improve their clinical use^39^. In our and previous studies evaluating blood-biomarker models^23–25^, including age and *APOE* ε4 status—known relevant risk factors of Aβ positivity^40,41^—led to more discriminative models with a higher spread in predictions, which can help in supporting better screening decisions, and such models will probably become more common in AD diagnostics. In other medical fields in which risk-prediction models are more frequently used, it is common to combine both condition-related biomarkers with other relevant variables, for example, risk factors and genetic information, such as the HEART score for identifying ischemic etiology of acute chest pain^21^ (combining demographics, risk factors and biomarkers of myocardial damage) and the STHLM3 model for diagnosing prostate cancer^22^ (combining demographics, genetic polymorphisms and prostate-specific antigen levels).

We acknowledge strengths and limitations of our study. A strength of the present study was that we included a large group of cognitively impaired participants, from three independent memory clinic cohorts from two geographically distinct settings. The workflow showed high performance in patients extensively phenotyped with two different plasma p-tau217 assay variants measured in different analytical platforms, two FDA-approved CSF Aβ42/Aβ40 assays and two Aβ-PET radiotracers. Taken together, we consider our design supports the potential generalizability of our findings, although further validation in diverse populations and settings is warranted. Although we first envision such a workflow to be applied in memory clinics with the capacity to handle advanced testing (CSF and/or PET) and new therapies, this workflow could be most useful in primary care in the future, possibly facilitating the referral process to specialist clinics. We highlight that the BioFINDER-1 and BioFINDER-2 populations in the present study consisted of memory clinic patients referred from primary care, presenting a wide range of comorbidities, and also presenting relatively low educational attainment (median 12 years) and similar age ranges to other aging and memory clinic cohorts^42–44^. Although the BioFINDER samples had higher proportions of men (and women who are more affected by AD), Aβ-PET positivity was more frequent in women (65.7%) than in men (57.3%). A limitation of our study is that plasma biomarker measurements, for each of the assays, were conducted in a single-batch manner (as is standard in cohort studies). Before clinical routine implementation, assays, cutoffs and biomarker-based model strategies will have to be prospectively validated. Another limitation is that the ideal reference standard for in silico evaluation of such a workflow would have been neuropathology, which is not yet available for the cohorts included, but our reference standard, Aβ-PET, has been widely validated against neuropathology^4^.

In conclusion, when screening patients with MCI for the presence of Aβ positivity, performing risk stratification with a plasma p-tau217-based model can lead to highly accurate classifications while substantially reducing the number of patients referred for further costly or invasive Aβ tests. Implementing such a workflow to detect AD in the future could considerably reduce advanced testing with CSF or PET, minimizing the burden for patients and caregivers, as well as the costs for healthcare providers.

## Methods

### Participants

In this cross-sectional study, we included patients with MCI from two independent cohorts, based on complete availability of plasma p-tau217, CSF Aβ42/Aβ40, Aβ-PET and *APOE* ε4 genotyping. Our model training cohort, BioFINDER-1 (NCT01208675), recruited patients between January 2010 and January 2015 and our validation cohort, BioFINDER-2 (NCT03174938), started recruitment in May 2017. In both cohorts, the patients were consecutively recruited from secondary memory clinics in the southern part of Sweden, where most of the study participants were referred directly from primary care, as described below. In Supplementary Information, we demonstrate that the included BioFINDER-1 and BioFINDER-2 populations (that is, with full biomarker availability) were similar to the nonincluded participants due to lack of data for one or more biomarkers (Supplementary Tables 7 and 8)^10,45,46^.

The BioFINDER-1 inclusion criteria for enrolling participants with subjective cognitive decline or MCI were as follows: (1) having been referred owing to cognitive symptoms experienced by the participant or perceived by an informant; (2) age between 60 and 80 years; (3) MMSE score of 24–30 points at the baseline visit; (4) do not fulfill the criteria for any dementia; and (5) fluency in Swedish. The exclusion criteria were as follows: (1) a systemic illness or organ failure of substantial severity that would hinder participation in the study; (2) current substance misuse or alcohol abuse; (3) refusal of neuropsychological assessment or lumbar puncture; and (4) cognitive impairment at baseline that could, with high confidence, be explained by another condition or disease, such as major cerebral hemorrhage, normal pressure hydrocephalus, brain tumor, brain infection, epilepsy, multiple sclerosis, psychotic disorders, severe depression or ongoing use of medication that causes a reduction in cognitive functioning (such as high-dose benzodiazepines). The clinical diagnosis was delivered at baseline based on an extensive battery of neuropsychological tests evaluating verbal and episodic memory, visuospatial ability and attention/executive domains, as described in detail elsewhere^46^. In the whole BioFINDER-1 study, for which enrollment was completed, a thorough analysis on referral origin had been previously conducted as described by Petrazzuoli et al.^46^. Most of the BioFINDER-1 patients (80.8%) were referred from primary care, whereas 12.5% of referrals were made by other specialist clinics and 6.7% of patients were self-referrals^46^. The inclusion criteria for recruitment of patients with MCI for BioFINDER-2 were as follows: (1) aged 40–100 years; (2) referred to the memory clinics due to cognitive symptoms; (3) MMSE score of 24–30 points; (4) did not fulfill the criteria for any dementia (major neurocognitive disorder) according to the *Diagnostic and Statistical Manual of Mental Disorders*, 4th edn (DSM-IV)^47^; and (5) fluent in Swedish. The BioFINDER-2 study also recruits patients who are CU, patients with AD dementia and patients with non-AD neurodegenerative conditions, and its general exclusion criteria were as follows: (1) unstable systemic illness that makes it difficult to participate in the study; (2) current alcohol or substance misuse; and (3) refusing lumbar puncture, MRI or PET. Out of the 212 MCI-included participants from BioFINDER-2 with readily available referral data, most were referred from primary care (*n* = 179; 84.4%), followed by hospital referrals (*n* = 31; 14.6%) and self-referrals (*n* = 2; 0.9%).

In both cohorts, a clinical diagnosis of MCI was made for those patients who did not meet the criteria for dementia (major cognitive disorder as in DSM-V^48^) but have lower scores than −1.5 s.d. in at least one cognitive domain such as memory, verbal, attention/executive or visuospatial function. In BioFINDER-1, a senior neuropsychologist made the diagnosis after a thorough neuropsychological battery to make this determination, as previously described^46^. In BioFINDER-2, the MCI diagnosis was based on a score <−1.5 *z*-scores in any cognitive domain, based on regression normative scores accounting for age, education and test performance in Aβ-negative controls^49^. The *z*-scores for each cognitive domain were calculated by averaging the *z*-scores of relevant tests, with further details on the derivation of such normative equations available elsewhere^50,51^. The domains included attention/executive function, verbal ability, memory and visuospatial function, and the tests used included Trail Making Test A, Trail Making Test B, Symbol Digit Modalities Test, verbal fluency animals, 15-word short version of the Boston Naming Test, 10-word delayed recall from the Alzheimer’s Disease Assessment Scale, and incomplete letters and cube analysis from the Visual Object and Space Perception battery.

In BioFINDER-1 and BioFINDER-2, we also evaluated the presence of comorbidities in the study population, evaluating for history of cardiovascular disease, diabetes or dyslipidemia^36^. Participants were considered to have cardiovascular disease if they presented with a history of either ischemic heart disease or hypertension, or if they were on anti-hypertensive/cardioprotective therapy. A history of dyslipidemia was considered when patients had such a diagnosis previously made or if they were on lipid-lowering therapy. Participants were considered to have CKD based on estimated glomerular filtration rate <60 ml min^−1^ per 1.73 m^2^, accepted as a functional criterion for CKD^52^.

In a secondary analysis, we included a subset of 84 cognitively impaired participants with available plasma p-tau217, CSF Aβ42/Aβ40, *APOE* ε4 genotype and Aβ-PET from the TRIAD cohort, recruited from a tertiary care memory clinic specializing in the diagnosis and management of neurodegenerative diseases^44^. All clinical diagnoses were made blinded to biomarker results. All participants had clinical assessments including Clinical Dementia Rating (CDR), MMSE and cerebrovascular disease risk using the Hachinski Ischemic Scale. Participants were excluded from the present study if they had systemic conditions that were not adequately controlled through a stable medication regimen. Other exclusion criteria were active substance abuse, recent head trauma, recent major surgery or MRI/PET safety contraindications. The included participants had MCI as defined based on a CDR of 0.5 and an MMSE between 24 and 30 (*n* = 63), and patients with dementia who had CDR of ≤1 (*n* = 21).

All BioFINDER and TRIAD patients gave their written informed consent to participate in the study and participation was voluntary. The BioFINDER studies were approved by the Ethical Review Board in Lund, Sweden, which is part of the Swedish Ethical Review Authority. TRIAD was approved by the Montreal Neurological Institute PET working committee and the Douglas Mental Health University Institute Research Ethics Board.

### Imaging and fluid biomarkers in BioFINDER-1 and BioFINDER-2

Aβ-PET was quantified using [^18^F]flutemetamol on a Philips Gemini TF 16 scanner in BioFINDER-1 and a digital GE Discovery MI scanner in BioFINDER-2. Scans were acquired 90–110 min after the injection of ~185 MBq of [^18^F]flutemetamol. The standardized uptake value ratio (SUVr) was obtained by normalizing the neocortical composite values to the whole cerebellum as a reference region. FreeSurfer (v.5.3) parcellation of the T1-weighted MR scan was used to transform the PET data to the participants’ native T1 space, so as to obtain mean regional SUVr values in predefined neocortical regions of interest, including prefrontal, lateral temporal, parietal, anterior cingulate and posterior cingulate/precuneus^53^. Aβ-PET data were binarized into normal and abnormal using cutoffs derived from Gaussian mixture modeling (GMM), with a threshold of ≥1.138 for BioFINDER-1 and ≥1.033 for BioFINDER-2.

CSF samples were collected and described based on previously described protocols^54^. CSF Aβ42/40 was measured using the fully automated Roche Elecsys NeuroTool Kit for the entirety of BioFINDER-1 and for 75% (*n* = 161) of BioFINDER-2 participants^55,56^. Abnormal CSF status was defined based on previously derived cutoffs determined using GMM, with a threshold of ≤0.066 for BioFINDER-1 and ≤0.080 for BioFINDER-2 (the higher cutoff in the latter study is due to use of LoBind tubes in BioFINDER-2, according to more recent protocols that prevent Aβ42 from binding to the tube walls^57,58^). For the 25% (*n* = 51) of BioFINDER-2 participants for whom the Elecsys measurements were not available, an abnormal CSF Aβ42/40 status was determined using the FDA-approved Lumipulse G assay, with a GMM-derived threshold of ≤0.06 (ref. ^59^). All CSF Aβ42/40 measurements were performed at the Clinical Neurochemistry Laboratory, Sahlgrenska Academy.

EDTA plasma samples were collected, handled and processed as previously described^10,45^. Plasma p-tau217 was quantified using the Mesoscale Discovery platform with an assay developed by Lilly Research Laboratories. Biotinylated-IBA493 was used as a capture antibody and SULFO-TAG-4G10-E2 (anti-tau) as the detector antibody, with sample and antibody dilution at 1:2, as previously described^23^. *APOE* ε4 was genotyped using a TaqMan allelic discrimination assay^60^.

### Imaging and fluid biomarkers in TRIAD

Individuals were evaluated with plasma p-tau217, CSF Aβ42/40 and amyloid-PET using [^18^F]AZD4694. Plasma concentrations of p-tau217 were measured using a Simoa assay developed by Janssen R&D by scientists blinded to clinical, demographic and biomarker information as described previously^16^, using the PT3 antibody as capture and HT43 as detector, and samples and detector were diluted 1:2. CSF concentrations of Aβ42/40 were quantified using the fully automated Lumipulse G1200 instrument (Fujirebio), with an Aβ-positivity threshold of 0.068, by scientists blinded to clinical and biomarker information as described previously^61^. A [^18^F]AZD4694 amyloid-PET-positivity threshold of 1.55 was employed (centiloid ≥ 24), validated based on GMM, CSF thresholds and visual assessments^62^. Blood and CSF collections took place on the same day.

### Statistics and reproducibility

First, we developed a logistic regression model using Aβ-PET status as the outcome with plasma p-tau217, age and *APOE* ε4 status as predictors in BioFINDER-1. Age and *APOE* ε4 were considered as predictors due to their inclusion in recently published, blood-based biomarker models and due to their well-described associations with Aβ positivity^23–25,40,41^. Plasma p-tau217 was log-transformed due to its skewed distribution and age was modeled with a linear term. Variables such as cognitive tests may be of more relevance to prognostic models (that is, predicting cognitive worsening) than in diagnostic models for Aβ positivity, given the poor association between Aβ load and symptoms^63^. To examine whether a simpler model would be preferred to this full model with age, *APOE* ε4 and p-tau217, backward variable deletion was performed during bootstrapped internal validation (*n* = 1,000), with the stopping criterion set at *α* = 0.157, recommended for model development scenarios such as ours^64^. The model most frequently chosen during this procedure was externally validated in BioFINDER-2. For model performance, we used the receiver operating characteristic’s AUC. In BioFINDER-1, the optimism-corrected AUC is reported, a metric recommended to account for overfitting-related optimism at model development^65^. Model calibration at external validation was assessed visually^66^. For goodness of fit, we report Nagelkerke’s pseudo-coefficient of determination (*R*^2^) and Akaike’s information criterion^65,67^.

Based on the blood biomarker, model-derived probabilities of Aβ-PET positivity and further testing with CSF Aβ42/Aβ40, we evaluated a two-step diagnostic workflow. In the first step, different thresholding strategies were explored to classify participants into low-, intermediate- and high-risk groups based on the plasma p-tau217 model-derived probabilities of Aβ-PET positivity. These strategies were defined based on lower probability thresholds with 90%, 95% and 97.5% sensitivity and higher probability thresholds with 90%, 95% and 97.5% specificity, with the same sensitivity and specificity always being tested together (for example, 90% sensitivity with 90% specificity). For each of the strategies, we calculated the prevalence of Aβ-PET negativity in the low-risk group along with the prevalence of Aβ-PET positivity in the high-risk group. For the second step, we tested the scenario in which further testing would be carried out with CSF Aβ42/Aβ40 measurements only in intermediate-risk participants from the first step. In this group, we reported the concordance between CSF and Aβ-PET status. Furthermore, we computed the overall workflow accuracy, represented by the proportion of correct Aβ-PET status classifications in both plasma and CSF steps, as well as the reduction in number of further confirmatory tests by the blood-biomarker-based risk stratification. In a secondary exploratory analysis, we further evaluated the robustness and generalizability of the two-step workflow using *z*-scored plasma p-tau217 values. The *z*-scores were obtained based on the distribution of this reference CU Aβ-negative sample as follows: (plasma p-tau concentration − mean p-tau concentration in CU Aβ negatives)/(s.d. of plasma p-tau concentration in CU Aβ-negatives). In BioFINDER-1, *z*-scored plasma p-tau217 (Lilly) values were obtained based on 283 CU Aβ-negative older adults with a mean (s.d.) plasma p-tau217 concentration of 0.153 (0.077) pg ml^−1^. In BioFINDER-2, based on 316 CU Aβ-negative participants, the mean (s.d.) concentrations were 0.156 (0.064) pg ml^−1^ for plasma p-tau217 (Lilly). In TRIAD, *z*-scores were calculated based on 111 Aβ-negative CU older adults with a mean (s.d.) plasma p-tau217 (Janssen) concentration of 0.052 (0.026) pg ml^−1^. Such a procedure enables application of the risk-prediction model for different plasma p-tau217 assays, because when *z*-scored they can be obtained from internal reference samples from clinical chemistry labs and memory clinic services. Briefly, the same original BioFINDER-1 model was re-fitted with *z*-scored plasma p-tau217 with the Lilly assay. Then, it was validated in two other cohorts: BioFINDER-2, based on *z*-scored Lilly plasma p-tau217 immunoassay and, in TRIAD, based on *z*-scored plasma p-tau217 measured with a different p-tau217 immunoassay (Janssen R&D). The whole workflow was re-evaluated for overall accuracy and reduction in the number of advanced tests for all of these secondary analyses, with the same risk thresholds from the original main analysis model. The *z*-scored model was developed in BioFINDER-1 in the exact same MCI population as that in the main analysis (*n* = 136). When validating the *z*-scored model in BioFINDER-2 with *z*-scored Lilly plasma p-tau217, we evaluated it in the exact same BioFINDER-2 MCI population as used in the main analysis (*n* = 212). In TRIAD, the *z*-scored model was applied in the *n* = 84 patients with cognitive impairment with key demographic characteristics shown in Supplementary Information. Our sample size was based on complete biomarker availability (for plasma, genetic, CSF and imaging data) rather than on statistically predetermined numbers, but our sample size was similar to those reported in previous publications evaluating risk-prediction models in AD^23–25^. When applicable, a two-sided *α* of 0.05 was used and 95% CIs are reported. No data exclusion was performed. Data collection and analysis were not randomized or performed blind to the experimental groups. All statistical analyses were performed in R v.4.1.1 (www.r-project.org), mainly using the ‘rms’ package^68^.

### Reporting summary

Further information on research design is available in the Nature Portfolio Reporting Summary linked to this article.

## Supplementary information

Supplementary Information Supplementary Tables 1–11.

Reporting Summary

## Data Availability

The present study does not include data available in external or online repositories. Anonymized data will be shared by request from a qualified academic investigator for the sole purpose of replicating procedures and results presented in the article. For BioFINDER, requests will be considered as long as data transfer is in agreement with EU legislation on the general data protection regulation and decisions by the Swedish Ethical Review Authority and Region Skåne, which should be regulated in a material transfer agreement and contact can be made through the study’s website (https://biofinder.se). Arrangements for data sharing for replication of the findings in the TRIAD dataset are subject to standard data-sharing agreements and further information can be found in the study’s website (https://triad.tnl-mcgill.com) or via direct contact with study leader pedro.rosa@mcgill.ca.

## Code availability

The code that supports the results of the present study is available from the corresponding authors upon request. All models were built using publicly available packages and functions in the R programming language.
