# Supplementary material for: A two-step workflow based on plasma p-tau217 to screen for amyloid β positivity with further confirmatory testing only in uncertain cases
Source: Nat Aging. 2023 Aug 31;3(9):1079–90. doi: 10.1038/s43587-023-00471-5 (PMC10501903; doi:10.1038/s43587-023-00471-5)
Supplement: Supplementary file 1 — Supplementary Tables 1–11. [file 43587_2023_471_MOESM1_ESM.pdf]

nature aging

Letter

https://doi.org/10.1038/s43587-023-00471-5

A two-step workflow based on plasma p-tau217 to screen for amyloid β positivity with further confirmatory testing only in uncertain cases

In the format provided by the authors and unedited

Table of Contents

Supplementary Table 1. BioFINDER-1 and BioFINDER-2 participant characteristics.

Supplementary Table 2. A full logistic regression model for Aβ-PET positivity is preferred at bootstrapped internal validation in BioFINDER-1.

Supplementary Table 3. Sensitivity analysis on different CSF biomarkers for determining Aβ-PET status in patients with intermediate risk.

Supplementary Table 4. Coefficients for the model for Aβ-PET-positivity developed in BioFINDER-1 with z-transformed plasma p-tau217 values .

Supplementary Table 5. Discriminative performance of the z-transformed plasma p-tau217 BioFINDER-1 model-derived probabilities in BioFINDER-2 and TRIAD.

Supplementary Table 6. Risk stratification for Aβ-PET positivity for the 95% Se/Sp strategy when applying the z-transformed model to different plasma p-tau217 assay variants in BioFINDER and TRIAD.

Supplementary Table 7. Participant characteristics for the TRIAD cohort.

Supplementary Table 8. Evaluating demographic differences according to classification status based on the 95% Se/Sp strategy based on the main analysis p-tau217 model.

Supplementary Table 9. Evaluating the accuracy of the 95% Se/Sp strategy when excluding patients with CKD.

Supplementary Table 10. Differences between included vs non-included participants in BioFINDER-1.

Supplementary Table 11. Differences between included vs non-included participants in BioFINDER-2.

Supplementary references

Supplementary Table 1. BioFINDER-1 and BioFINDER-2 participant characteristics.

	BioFINDER-1 (n = 136)	BioFINDER-2 (n = 212)	Combined cohorts (n = 348)
Age, years	72.0 (67.7-75.8)	73.2 (68.6-77.0)	73.0 (68.3-76.5)
Sex, female n (%)	48 (35.3)	89 (42.0)	137 (39.4)
Education, years	11.0 (9.0-13.0)	12.0 (9.0-16.0)	12.0 (9.0-14.0)
MMSE score	27.0 (26.0-29.0)	27.0 (26.0-29.0)	27.0 (26.0-29.0)
APOE ε4 carriers, n (%)	67 (49.3)	117 (55.2)	184 (52.9)
Aβ-PET positive, n (%)	82 (60.3)	129 (60.8)	211 (60.6)
CSF Aβ42/Aβ40 positive, n (%)	87 (64.0)	126 (59.4)	213 (61.2)
Plasma p-tau217, pg/mL	0.27 (0.18-0.43)	0.26 (0.15-0.44)	0.27 (0.16-0.44)
Comorbidities*			
Cardiovascular disease, n (%)	75 (55.1%)	113 (53.3%)	188 (54.0%)
Diabetes, n (%)	19 (14.0%)	36 (17.0%)	55 (15.8%)
Dyslipidemia, n (%)	49 (36.0%)	83 (39.2%)	132 (37.9%)
eGFR, mL/min/m^2	77.2 (67.7-85.9)	82.4 (69.1-88.4)	79.2 (68.0-87.8)
Chronic kidney disease, n (%)	15 (11.0%)	17 (8.0%)	32 (9.2%)

Data are n; median (Q1-Q3) or n (%). The participant characteristics are shown for BioFINDER-1 (model training cohort), BioFINDER-2 (model validation cohort) and for both cohorts combined. Aβ-PET status was determined based on previously described thresholds. Aβ = Amyloid-β. SUVr = Standardized uptake value ratio. P-tau217 = tau phosphorylated at threonine 217. MMSE = Mini-Mental State Examination. PET = positron

emission tomography. *APOE* ε4 = apolipoprotein E allele ε4. eGFR = estimated glomerular filtration rate. * Among BioFINDER-1 participants, eGFR (and thus CKD) data was not available for a small fraction of participants (n=9, 6.6%), and was also missing in BioFINDER-2 for roughly one third of participants (n=64, 30%). Among BioFINDER-2 participants, data on medical history and medication use was not available for a small fraction of the participants (n=12, 3.4%).

Supplementary Table 2. A full logistic regression model for Aβ-PET positivity is preferred at bootstrapped internal validation in BioFINDER-1.

	Predictor coefficients, p-value						Likelihood ratio test *vs* full model		Frequency of selection at backward variable deletion, %
**Model**	**P-tau217**	**Age**	***APOE* ε4 status**	**R^2**	**AIC**	**AUC, %**	**X^2**	**P-value**	
P-tau217 + Age + *APOE* ε4 status	1.84, p<0.0001	0.08, p=0.084	1.8, p=0.0003	0.561	118	89.3%	72.9	-	63.4%
P-tau217	2.12, p<0.0001	-	-	0.448	132	84.7%	54.7	<0.0001	0.5%
Age	-	0.08, p=0.0117	-	0.065	180	61.7%	6.7	<0.0001	0%
APOE ε4 status	-	-	2.13, p<0.0001	0.280	155	74.0%	31.5	<0.0001	0%
P-tau217 + Age	2.06, p<0.0001	0.08, p=0.0592	-	0.473	130	85.4%	58.4	<0.001	0.4%
P-tau217 + *APOE* ε4 status	1.95, p<0.0001	-	1.95, p=0.002	0.543	119	88.2%	69.8	0.0837	35.7%
Age + *APOE* ε4 status	-	0.1, p=0.009	2.23, p<0.0001	0.336	150	79.6%	38.9	<0.0001	0%

Starting from a full screening model for Aβ-PET positivity including plasma p-tau217, age and *APOE* ε4 status, backward variable selection was performed at internal validation (n=1000 bootstrap samples) during model training in BioFINDER-1. Deleting predictors from the model led to the full model plus other six model candidates, indicated in the first column. Regression coefficients are shown for p-tau217 (log2 transformed), age, and *APOE* ε4 carriership for each of the models. Goodness-of-fit metrics R^2 and AIC are shown alongside the AUC for discrimination. Likelihood ratio tests were performed between the full model and each of the model candidates, with chi-squared (X^2) statistics and p-values shown for each test. At each bootstrap procedure, model terms were deleted from the full model based on not meeting α=0.157 at two-sided likelihood ratio tests,

with no multiple testing adjustment. This constitutes backward variable selection, i.e. removing terms from a full model with all predictors (the full model is the reference) rather than sequentially including predictors, and is more recommended for model development[1]. The last column indicates the frequency that each model was selected across all the bootstrap procedures, with the full model being selected 63.4% of the times and thus being selected as the model for external validation in BioFINDER-2. The intercept coefficient for this full model was -2.32 (not included in the table as was not relevant to model selection but is necessary to compute individual level probabilities). Aβ = Amyloid-β. PET = Positron emission tomography. P-tau217 = tau phosphorylated at threonine 217. *APOE* ε4 = Apolipoprotein E allele ε4. AUC = area under the curve. R^2 = coefficient of determination. AIC = Akaike's information criterion.

Supplementary Table 3. Sensitivity analysis on different CSF biomarkers for determining Aβ-PET status in patients with intermediate risk.

CSF Biomarker	Risk stratification strategy	Intermediate-risk group size, n	Aβ-PET status, n, negative/positive	PPA, %	NPA, %	OPA, %
	Se/Sp 90%	40	20/20	80.0%	100%	87.0%
Aβ42/Aβ40	Se/Sp 95%	85	35/50	83.9%	89.7%	85.9%
	Se/Sp 97.5%	120	55/65	85.5%	88.2%	86.7%
	Se/Sp 90%	40	20/20	75.0%	75.0%	75.0%
Aβ42	Se/Sp 95%	85	35/50	79.1%	61.9%	70.6%
	Se/Sp 97.5%	120	55/65	78.2%	66.2%	71.7%
	Se/Sp 90%	40	20/20	100%	76.9%	75.0%
P-tau181/Aβ42	Se/Sp 95%	85	35/50	97.2%	69.4%	81.1%
	Se/Sp 97.5%	120	55/65	95.7%	71.6%	80.8%

The table describes the performance of different CSF biomarkers for determining Aβ-PET status in step-2 for the group of MCI individuals classified as intermediate-risk at step-1 based on all risk stratification thresholding strategies evaluated, (Se/Sp 90, 95 and 97.5%), based on the subset of BioFINDER-1 and BioFINDER-2 participants with available CSF Elecsys biomarkers. Cutoffs for Aβ42/Aβ40 were as described in Supplementary Methods 3. For CSF p-tau181/Aβ42 and Aβ42, cutoffs of ≥0.023 and ≤1030 pg/mL were used, respectively, as previously described[2]. Aβ42/Aβ40 is the biomarker with the most balanced performance in this group of patients with uncertain outcomes, with OPAs (overall percent agreement) ranging from 85.9-87.0% depending on the risk stratification strategy. CSF Aβ42 alone performed on a lower-tier, with OPA's ranging from 70.6-75.0%. CSF P-tau181/Aβ42 demonstrated intermediate OPA's (75.0-81.1%) among the three evaluated markers, but with higher PPA's (95.7-100%). OPA = overall percent agreement. PPA = positive percent agreement. NPA = negative percent agreement. CSF = cerebrospinal fluid. Aβ = Amyloid-β. PET = Positron emission tomography. P-tau181 = tau phosphorylated at threonine 181. Se = Sensitivity. Sp = Specificity.

Supplementary Table 4. Coefficients for the model for Aβ-PET-positivity developed in BioFINDER-1 with z-transformed plasma p-tau217 values.

Model term	Coefficient	Standard Error	P-value
Intercept	-6.98	3.18	0.03
P-tau217, z-transformed	0.605	0.14	<0.0001
APOE ε4 status	1.80	0.48	0.0003
Age	0.077	0.04	0.0837

Table indicating the coefficients of the re-fitted BioFINDER-1 model for Aβ-PET positivity using plasma p-tau217 z-transformed concentrations based on a reference sample of CU Aβ-negative individuals, with two-sided p-values reported for each regression term at an alpha=0.05 and no multiplicity adjustment. These were the coefficients used to extract probabilities for BioFINDER-2 in the secondary analyses with p-tau217 assay variants, as well as in TRIAD. Aβ = Amyloid-β. PET = Positron emission tomography. *APOE* ε4 = apolipoprotein E allele ε4. CU = cognitively unimpaired. P-tau217 = tau phosphorylated at threonine 217. AUC = Area under the curve.

Supplementary Table 5. Discriminative performance of the z-transformed plasma p-tau217 BioFINDER-1 model-derived probabilities in BioFINDER-2 and TRIAD.

Model phase	Cohort	Sample size, n	Plasma biomarker	Technique, assay	AUC, %	95% CI
Development	BioFINDER-1	136	P-tau217	Immunoassay, Lilly	88.6	82.9-94.2
Validation	BioFINDER-2	212	P-tau217	Immunoassay, Lilly	93.9	90.9-96.9
	TRIAD	84	P-tau217	Immunoassay, Janssen	87.1	78.5-95.6

The table shows the discriminative ability of the predicted probabilities of Aβ-PET-positivity, calculated based on the coefficients from the z-transformed plasma p-tau217 developed in BioFINDER-1, as described in the previous Supplementary Table. The table shows that when using plasma p-tau217 concentrations z-transformed to a reference CU Aβ-negative, inter-assay application of a risk prediction model is feasible, leading to highly discriminative risk predictions regardless of which assay or p-tau217 variant the model is developed with or applied to. Aβ = Amyloid-β. PET = Positron emission tomography. AUC = area under the receiver operating characteristic curve. CU = cognitively unimpaired. P-tau217 = tau phosphorylated at threonine 217.

Supplementary Table 6. Risk stratification for Aβ-PET positivity for the 95% Se/Sp strategy when applying the z-transformed model to different plasma p-tau217 assay variants in BioFINDER and TRIAD.

Risk groups	Participants in each risk group, n	Within-risk group Aβ-PET status	
		Aβ-PET negative, n, %	Aβ-PET positive, n, %
BioFINDER-1, model development Z-scored Lilly plasma p-tau217 (n=136)			
Low risk (<31)	35	29 (82.9%)	6 (17.1%)
Intermediate risk (31-80)	44	21 (47.7%)	23 (52.3%)
High risk (>80)	57	4 (7.0%)	53 (93.0%)
BioFINDER-2, model validation Z-scored Lilly plasma p-tau217, (n=212)			
Low risk (<31)	59	54 (91.5%)	5 (8.5%)
Intermediate risk (31-80)	61	25 (41.0%)	36 (59.0%)
High risk (>80)	92	4 (4.3%)	88 (95.7%)
TRIAD, model validation Z-scored Janssen plasma p-tau217, (n=84)			
Low risk (<31)	24	20 (83.3%)	4 (16.7%)
Intermediate risk (31-80)	27	11 (40.7%)	16 (59.3%)
High risk (>80)	33	2 (6.1%)	31 (93.9%)

Data are presented as n or n (%). The first column indicates the cohort and plasma p-tau217 assay variant used when evaluating the risk stratification performance of the 95% Se/Sp threshold strategy for probabilities obtained with the z-transformed BioFINDER-1 model, developed with the p-tau217 assay from Lilly used in main analyses. In BioFINDER-2, these analyses were done with predicted probabilities obtained using the same assay version as in BioFINDER-1, with a different assay being using in TRIAD. The same thresholds derived in the original main analysis with the concentration based p-tau217 BioFINDER-1 model were used. For each of the assay-cohort combinations (rows in first column), the second column corresponds to the number of individuals falling in each risk category. Lastly, Aβ-status is shown for low-, intermediate- and high-risk groups. The % of Aβ-negatives in the low-risk group and the

% of Aβ-positives in the high risk group corresponds to each evaluated thresholds' NPV and PPV, respectively. NPV = Negative predictive value. PPV = Positive predictive value. Aβ = Amyloid-β. PET = Positron emission tomography. P-tau217 = tau phosphorylated at threonine 217. Se = Sensitivity. Sp = Specificity.

Supplementary Table 7. Participant characteristics for the TRIAD cohort.

	TRIAD (n = 84)
Age, years	68.3 (7.95)
Sex, female n (%)	46 (54.8)
Education, years	15.1 (3.42)
MMSE score	25.4 (5.1)
Diagnosis, MCI/dementia, n	63/21
APOE ε4 carriers, n (%)	39 (46)
Aβ-PET positive, n (%)	51 (60.7)
CSF Aβ42/Aβ40 positive, n (%)	54 (64)
Plasma p-tau217, pg/mL	0.153 (0.12)
Plasma p-tau217, z-transformed	4.71 (5.72)

Data are n (%) or mean (SD). The table summarises key demographic information of the n=84 cognitively impaired participants included from the TRIAD cohort for the secondary validation analyses. Aβ = Amyloid-β. PET = Positron emission tomography. *APOE* ε4 = apolipoprotein E allele ε4. CSF = cerebrospinal fluid. P-tau217 = tau phosphorylated at threonine 217. MCI = mild cognitive impairment.

Supplementary Table 8. Evaluating demographic differences according to classification status based on the 95% Se/Sp strategy based on the main analysis p-tau217 model.

	True-negatives (Low-risk, Aβ-; n=89, 25.6%)	False-negatives (Low-risk, Aβ+; n=11, 3.2%)	True-positives (High-risk, Aβ+; n=140, 40.2%)	False-positives (High-risk, Aβ-; n=7, 2.0%)	Intermediate risk (n= 101, 29.0%)	Overall (n=348)
Age, years	69.4 (62.5-73.8)	72.3 (68.4-73.9)	73.8 (69.0-77.1)	77.3 (70.3-76.9)	73.8 (70.3-76.9)	73.0 (68.3-76.5)
Sex, female n (%)	29 (32.6%)	4 (36.4%)	61 (43.6%)	2 (28.6%)	41 (40.6%)	137 (39.4%)
Education, years	12.0 (9.0-14.0)	9.50 (7.3-16.0)	12.0 (9.0-15.0)	9.00 (8.0-11.5)	12.0 (900-14.6)	12.0 (9.0-14.0)
MMSE score	28.0 (27-29)	28.0 (26-29)	27 (25-28)	26 (24-27)	28 (26-29)	27 (26-29)
APOE ε4 carriers, n (%)	10 (11.2%)	2 (18.2%)	125 (89.3%)	5 (71.4%)	42 (41.6%)	184 (52.9%)
Aβ-PET positive, n (%)	0 (0%)	11 (100%)	140 (100%)	0 (0%)	64 (63.4%)	213 (61.2%)
CSF Aβ42/Aβ40 positive, n (%)	1 (1.1%)	7 (63.6%)	138 (98.6%)	3 (42.8%)	60 (59.4%)	211 (60.6%)
Plasma p-tau217, pg/mL	0.131 (0.101-0.163)	0.158 (0.129-0.191)	0.467 (0.345-0.626)	0.332 (0.278-0.447)	0.240 (0.195-0.300)	0.266 (0.163-0.438)
Comorbidities						
Cardiovascular disease, n (%)	48 (53.9%)	6 (54.5%)	62 (44.3%)	4 (54.5%)	68 (67.3%)	188 (54.0%)
Diabetes, n (%)	15 (16.9%) n=4 missing	2 (18.2%)	16 (11.4%) n=6 missing	0 n=1 missing	22 (21.8%) n=1 missing	55 (15.8%)
Dyslipidemia, n (%)	32 (36.0%)	4 (36.4%)	48 (34.3%)	2 (28.6%)	46 (45.5%)	132 (37.9%)
eGFR, mL/min/1.73m^2	84.6 (73.6-92.1) n=17 missing	86.1 (73.3-89.3) n=4 missing	79.0 (68.7-85.4) n=29 missing	57.1 (53.9-66.8) n=1 missing	76.5 (65.7-86.3) n=22 missing	79.1 (68.0-87.7)
CKD (eGFR<60)	5 (5.6%) n=17 missing	1 (9.1%) n=4 missing	12 (8.6%) n=29 missing	4 (57.1%) n=1 missing	10 (9.9%) n=22 missing	32 (9.2%)

Data are n; median (Q1-Q3) or n (%). The table describes key BioFINDER-1 and BioFINDER-2 MCI participant characteristics according to their classification status at the blood biomarker risk stratification step (step-1) based on the 95% Se/Sp probability thresholding strategy and with the

main analysis Lilly plasma p-tau217 model. Patients are stratified into true-negatives (low-risk label at step-1 who were also Aβ-PET-negative), false-negatives (low-risk label at step-1 who were Aβ-PET-positive), true-positives (high-risk label at step-1 who were also Aβ-PET-positive), false-positives (high-risk label at step-1 who were Aβ-PET-negative), intermediate-risk (assumed to be referred for a CSF test). Missing data are indicated when applicable. Most importantly, false-negatives had low p-tau217 values than expected and false-positives had higher p-tau217 values than expected. eGFR was reduced in the false-positive group, but the lower number of individuals in these group and the presence of incorrect classifications across all normal and abnormal eGFR span (shown in Supplementary Figure 4) indicates this might be of limited clinical relevance. Aβ = Amyloid-β. PET = Positron emission tomography. eGFR = estimated glomerular filtration rate. *APOE* ε4 = apolipoprotein E allele ε4. CSF = cerebrospinal fluid. CKD = chronic kidney disease. P-tau217 = tau phosphorylated at threonine 217. Se = Sensitivity. Sp = Specificity.

Supplementary Table 9. Evaluating the accuracy of the 95% Se/Sp strategy when excluding patients with CKD.

	Original population, regardless of eGFR availability (n=348)	Population with available eGFR, no eGFR-based exclusion, (n=275)	Population with available eGFR, excluding eGFR<60, (n=243)
Low-risk, n (%)	100 (28.7%)	79 (28.7%)	73 (30.0%)
Correctly classified (Aβ PET-), n (%)	89 (89.0%)	72 (91.1%)	67 (91.8%)
False-negatives, n (%)	11 (11.0%)	7 (8.9%)	6 (8.2%)
Intermediate-risk, n (%)	101 (29.0%)	79 (28.7%)	69 (28.4%)
High-risk, n (%)	147 (42.2%)	117 (42.5%)	101 (41.6%)
Correctly classified (Aβ PET+), n (%)	140 (95.2%)	111 (94.9%)	99 (98.0%)
False-positives, n (%)	7 (4.8%)	6 (5.1%)	2 (2.0%)

The table describes the risk stratification accuracy for the BioFINDER-1 and BioFINDER-2 MCI participants according to their classification status at the 95% Se/Sp probability thresholding strategy with the main analysis Lilly plasma p-tau217 model. In the first column, accuracy is reported regardless of eGFR availability. In the second column, only those patients with available eGFR are included, for a more clear comparison. In the third column, accuracy is displayed for the scenario in which patients with reduced eGFR would be excluded. In short, excluding these patients this would lead to a lower false-positive rate (from 4.8-5.1% to 2.0%), with its clinical impact further explored in the main text and other supplementary material. Aβ = Amyloid-β. PET = Positron emission tomography. eGFR = estimated glomerular filtration rate. *APOE* ε4 = apolipoprotein E allele ε4. CSF = cerebrospinal fluid. CKD = chronic kidney disease. P-tau217 = tau phosphorylated at threonine 217. Se = Sensitivity. Sp = Specificity.

Supplementary Table 10. Differences between included vs non-included participants in BioFINDER-1.

BioFINDER-1	Not included (missing CSF, PET, or both); n=128)	Included (complete biomarker availability; n=136)	p-value	Overall (n = 264)
Age, years	72.4 (68.3-76.2)	72.0 (67.7-75.8)	0.73	72.3 (67.9-76.1)
Sex, female n (%)	60 (46.9%)	48 (35.3%)	0.08	108 (40.9%)
Education, years	11.0 (8.00-14.0)	11.0 (9.00-13.0)	0.74	11.0 (8.00-13.0)
MMSE score	27.0 (25.0-29.0)	27.0 (26.0-29.0)	0.07	27.0 (26.0-29.0)
APOE ε4 carriers, n (%)	66 (51.6%)	67 (49.3%)	0.80	133 (50.4%)
Aβ-PET positive, n (%)	10 (55.6%); 110 missing	82 (60.3%)	0.89	92 (59.7%)
CSF Aβ42/Aβ40 positivity, n (%)	62 (57.4%); 11 missing	87 (64.0%)	0.36	149 (61.1%)
Plasma p-tau217, pg/mL	0.263 (0.190-0.416)	0.271 (0.180-0.427)	0.90	0.266 (0.184-0.421)
Comorbidities				
Cardiovascular disease, n (%)	65 (50.8%)	75 (55.1%)	0.56	140 (53.0%)
Diabetes, n (%)	6 (4.7%)	19 (14.0%)	0.03	25 (9.5%)
Dyslipidemia, n (%)	44 (34.4%)	49 (36.0%)	0.88	93 (35.2%)
eGFR, mL/min/1.73m^2	79.7 (69.7-88.1); 6 missing	77.2 (67.7-85.9) 9 missing	0.20	78.1 (68.9-86.8)
Reduced renal function, n (%)	12 (9.8%); 6 missing	15 (11.8%) 9 missing	0.77	27 (10.8%)

Data are n; median (Q1-Q3) or n (%). The table described key characteristics among the BioFINDER-1 MCI participants who had available plasma p-tau217 and were initially eligible for this study, stratified into columns according to whether they did or did not have complete availability of plasma p-tau217, *APOE* ε4 genotype, CSF Aβ42/Aβ40 and Aβ-PET. All of the data considered was for the baseline visit, with no lag or mismatches between biomarker collection. Out of the whole initially eligible MCI population (n=264), 136 patients fulfilled the inclusion criteria and 128 did not. Demographic characteristics were generally similar for included and non-included participants. Continuous variables were compared with t-tests and categorical variables with Chi-squared tests, at a two-sided alpha of 0.05, and no multiplicity adjustment was done. MCI = mild cognitive impairment Aβ = amyloid-β. CSF = cerebrospinal fluid. P-tau217 = tau phosphorylated at threonine 217. MMSE = Mini-Mental State Examination. PET = positron emission

tomography. *APOE* ε4 = apolipoprotein E allele ε4. eGFR = estimated glomerular filtration rate. Missing data are indicated within the table when applicable.

Supplementary Table 11. Differences between included vs non-included participants in BioFINDER-2.

BioFINDER-2	Not included (incomplete biomarker availability; n=34)	Included (complete biomarker availability; n=212)	p-value	Overall (n = 246)
Age, years	72.2 (69.4-75.6)	73.2 (68.6-77.0)	0.79	73.2 (68.6-76.7)
Sex, female n (%)	13 (39.4%)	89 (42.0%)	0.83	102 (41.5%)
Education, years	12.0 (9.0-15.0)	12.0 (9.00-16.0)	0.50	12.0 (9.00-16.0)
MMSE score	27.0 (25.0-29.0)	27.0 (26.0-29.0)	0.59	27.0 (26.0-29.0)
APOE ε4 carriers, n (%)	17 (50.0%)	117 (55.2%)	0.71	134 (54.5%)
Aβ-PET positive, n (%)	8 (72.7%); 23 missing	129 (60.8%)	0.64	137 (55.7%)
CSF Aβ42/Aβ40 positivity, n (%)	16 (69.6%); 11 missing	126 (59.4%)	0.47	142 (57.7%)
Plasma p-tau217, pg/mL	0.341 (0.192-0.426)	0.246 (0.151-0.439)	0.28	0.270 (0.160-0.438)
Comorbidities				
Cardiovascular disease, n (%)	18 (52.9%)	113 (53.3%)	0.99	131 (53.3%)
Diabetes, n (%)	6 (17.6%); 3 missing	36 (17.0%); 12 missing	0.98	42 (17.1%)
Dyslipidemia, n (%)	15 (44.1%)	83 (39.2%)	0.72	98 (39.8%)
eGFR, mL/min/1.73m^2	82.3 (72.9-87.9); 14 missing	82.1 (68.9-88.4); 64 missing	0.92	82.2 (69.2-88.4)
Reduced renal function, n (%)	3 (15.0%); 14 missing	17 (11.5%); 64 missing	0.93	20 (11.9%)

Data are n; median (Q1-Q3) or n (%). The table described key characteristics among the BioFINDER-2 MCI participants who had available plasma p-tau217 and were initially eligible for this study, stratified into columns according to whether they did or did not have complete availability of plasma p-tau217, *APOE* ε4 genotype, CSF Aβ42/Aβ40 and Aβ-PET. All of the data considered was for the baseline visit, with no lag or mismatches between biomarker collection. Out of the whole initially eligible MCI population (n=246), 212 patients fulfilled the inclusion criteria and 34 did not. Demographic characteristics were generally similar for included and non-included participants. Continuous variables were compared with t-tests and categorical variables with Chi-squared tests, at a two-sided alpha of 0.05, and no multiplicity adjustment was done. MCI = mild cognitive impairment Aβ = amyloid-β. CSF = cerebrospinal fluid. P-tau217 = tau phosphorylated at threonine 217. MMSE = Mini-Mental State Examination. PET = positron emission

tomography. *APOE* ε4 = apolipoprotein E allele ε4. eGFR = estimated glomerular filtration rate. Missing data are indicated within the table when applicable

eReferences

1. Steyerberg EW, Eijkemans MJC, Harrell Jr FE, Habbema JDF. Prognostic modelling with logistic regression analysis: a comparison of selection and estimation methods in small data sets. *Stat Med*. 2000;19(8):1059-1079. doi:10.1002/(SICI)1097-0258(20000430)19:8<1059::AID-SIM412>3.0.CO;2-0

2. Blennow K, Stomrud E, Zetterberg H, et al. Second-generation Elecsys cerebrospinal fluid immunoassays aid diagnosis of early Alzheimer's disease. *Clin Chem Lab Med CCLM*. 2023;61(2):234-244. doi:10.1515/cclm-2022-0516
