# Supplementary material for: A two-step workflow based on plasma p-tau217 to screen for amyloid β positivity with further confirmatory testing only in uncertain cases
Source: Nat Aging. 2023 Aug 31;3(9):1079–90. doi: 10.1038/s43587-023-00471-5 (PMC10501903; doi:10.1038/s43587-023-00471-5)
Supplement: Supplementary file 2 — Reporting Summary [file 43587_2023_471_MOESM2_ESM.pdf]

nature portfolio

Corresponding author(s): Oskar Hansson, Wagner S. Brum

Last updated by author(s): Jul 7, 2023

Reporting Summary

Nature Portfolio wishes to improve the reproducibility of the work that we publish. This form provides structure for consistency and transparency in reporting. For further information on Nature Portfolio policies, see our Editorial Policies and the Editorial Policy Checklist.

Statistics

For all statistical analyses, confirm that the following items are present in the figure legend, table legend, main text, or Methods section.

n/a	Confirmed	
☐	☒	The exact sample size (n) for each experimental group/condition, given as a discrete number and unit of measurement
☐	☒	A statement on whether measurements were taken from distinct samples or whether the same sample was measured repeatedly
☐	☒	The statistical test(s) used AND whether they are one- or two-sided<br>*Only common tests should be described solely by name; describe more complex techniques in the Methods section.*
☐	☒	A description of all covariates tested
☐	☒	A description of any assumptions or corrections, such as tests of normality and adjustment for multiple comparisons
☐	☒	A full description of the statistical parameters including central tendency (e.g. means) or other basic estimates (e.g. regression coefficient) AND variation (e.g. standard deviation) or associated estimates of uncertainty (e.g. confidence intervals)
☐	☒	For null hypothesis testing, the test statistic (e.g. F, t, r) with confidence intervals, effect sizes, degrees of freedom and P value noted<br>*Give P values as exact values whenever suitable.*
☒	☐	For Bayesian analysis, information on the choice of priors and Markov chain Monte Carlo settings
☒	☐	For hierarchical and complex designs, identification of the appropriate level for tests and full reporting of outcomes
☒	☐	Estimates of effect sizes (e.g. Cohen's d, Pearson's r), indicating how they were calculated

Our web collection on statistics for biologists contains articles on many of the points above.

Software and code

Policy information about availability of computer code

Data collection	No software was used.
Data analysis	All statistical analyses were performed in R version 4.1.1 (www.r-project.org), with regression analyses done with the "rms" package (version 6.1.0).

For manuscripts utilizing custom algorithms or software that are central to the research but not yet described in published literature, software must be made available to editors and reviewers. We strongly encourage code deposition in a community repository (e.g. GitHub). See the Nature Portfolio guidelines for submitting code & software for further information.

Data

Policy information about availability of data

All manuscripts must include a data availability statement. This statement should provide the following information, where applicable:

- Accession codes, unique identifiers, or web links for publicly available datasets
- A description of any restrictions on data availability
- For clinical datasets or third party data, please ensure that the statement adheres to our policy

This study does not include data available in external or online repositories. Anonymized data will be shared by request from a qualified academic investigator for the sole purpose of replicating procedures and results presented in the article. For BioFINDER, requests will be considered as long as data transfer is in agreement with EU legislation on the general data protection regulation and decisions by the Swedish Ethical Review Authority and Region Skåne, which should be regulated in

a material transfer agreement, and contact can be made through the study's website (https://biofinder.se/). Arrangements for data sharing for replication of the findings in the TRIAD data set are subject to standard data-sharing agreements, and further information can be found in the study's website (https://triad.tnl-mcgill.com/).

Human research participants

Policy information about studies involving human research participants and Sex and Gender in Research.

Reporting on sex and gender	We used the term "sex" in the manuscript, and sex was determined based on participant self-reporting. The cohorts included recruit both self-reported men and women.
Population characteristics	Detailed information on demographics from the two cohorts, BioFINDER-1 and BioFINDER-2, is provided in Table 1. Participants had mild cognitive impairment (MCI) and had available data for biomarkers relevant for this study (Aβ-PET, CSF Aβ42/Aβ40, plasma p-tau217, APOE genotype). Overall, the study population (n=348) had a median (IQR) age of 73 (63.-76.5) years, 39.4% of participants were female, 52.9% were APOEe4 carriers, and median (IQR) years of educational attainment were 12 (9-14). Common comorbidities in older adults were also present, such as cardiovascular disease (54.0%), diabetes (15.8%), and dyslipidemia (37.9%). In TRIAD, participants were slightly younger [mean (SD) age 68 (8) years], with slightly more females (n=46; 55%), with key demographic characteristics summarised in Supplementary Table 6.
Recruitment	BioFINDER-1 (NCT01208675) and BioFINDER-2 (NCT03174938) are memory clinic based cohorts recruiting participants forwarded from primary care to secondary memory clinics in southern Sweden due to cognitive complaints. BioFINDER-1 participants were recruited between January 2010 and January 2015. BioFINDER-2 started recruitment in May 2017. TRIAD is a cohort enrolling patients mostly from memory clinic settings, as well as voluntaries. In both cohorts, memory clinic patients are invited by their care providers to participate in the study. The potential bias of these recruitment strategies is that the findings will be more restricted to memory patients, with a limited generalizability to primary care. It is discussed in the manuscript that such primary care validation is warranted by further studies.
Ethics oversight	All BioFINDER and TRIAD patients gave their written informed consent to participate in the study, and participation was voluntary. The BioFINDER studies were approved by the Ethical Review Board in Lund, Sweden, which is part of the Swedish Ethical Review Authority (BioFINDER-1: 2010-156; BioFINDER-2: 2016-1053). TRIAD was approved by the Montreal Neurological Institute PET working committee and the Douglas Mental Health University Institute Research Ethics Board (IUSMD16-61, IUSMD16-60).

Note that full information on the approval of the study protocol must also be provided in the manuscript.

Field-specific reporting

Please select the one below that is the best fit for your research. If you are not sure, read the appropriate sections before making your selection.

☒ Life sciences ☐ Behavioural & social sciences ☐ Ecological, evolutionary & environmental sciences

For a reference copy of the document with all sections, see nature.com/documents/nr-reporting-summary-flat.pdf

Life sciences study design

All studies must disclose on these points even when the disclosure is negative.

Sample size	Our development cohort (BioFINDER-1: n=136) and validation cohorts (BioFINDER-2: n=212; TRIAD: n=84) were cross-sectionally analyzed based on complete biomarker availability for plasma, genetic, cerebrospinal fluid and imaging data. There is no indication that we were insufficiently powered for these analyses considering a potential predictive utility of these algorithms. Obtaining such a number of individuals with such a comprehensive biomarker panel is a strength of the study, and similar clinical prediction modeling AD studies, that included patients with a more restrict biomarker panel, had roughly similar numbers (Verberk et al Ann Neurol, 2018 Nov;84(5):648-658; Janelidze et al Alzheimers Dement, 2022 Feb;18(2):283-293; Hu et al, JAMA Netw Open, 2022 Apr 1;5(4):e228392).
Data exclusions	The main inclusion criteria was that participants presented cognitive impairment, relevant clinical phenotype for current detection of AD pathology for considering potential novel treatments. Also, participants needed to have available data, for the same baseline visit, for plasma p-tau217, cerebrospinal fluid Aβ42/Aβ40, and Aβ-PET. Both BioFINDER-1 and BioFINDER-2 have recruited participants with MCI and dementia. However, per protocol in both cohorts, Aβ-PET is not carried in the later clinical stage of dementia. Thus, our analyses focused on the MCI population with the required biomarkers for testing our hypothesis. Within the eligible participants, no datapoint or individual was excluded from any analyses.
Replication	When developing a prediction model, such as we did in BioFINDER-1, validating it externally (i.e. obtaining risk probabilities using the coefficients from the developed model) it is crucial to determine its potential utility. The model developed in BioFINDER-1 demonstrated to have validated very well in BioFINDER-2, displaying very high discrimination and good calibration, with this validation being tested only once. For the geographical validation, the model also validated well, and an independent analyst (JT) carried data analyses, with validation being performed only once as well.
Randomization	No allocation into experimental groups was performed. Thus, randomization is not relevant to this study.

Blinding

All plasma, CSF and PET analyses were performed by individuals who were blinded to the clinical data. Authors who performed the data pre-processing were blinded to demographic and clinical characteristics of individuals.

Reporting for specific materials, systems and methods

We require information from authors about some types of materials, experimental systems and methods used in many studies. Here, indicate whether each material, system or method listed is relevant to your study. If you are not sure if a list item applies to your research, read the appropriate section before selecting a response.

Materials & experimental systems

n/a	Involved in the study	
☐	☒	Antibodies
☒	☐	Eukaryotic cell lines
☒	☐	Palaeontology and archaeology
☒	☐	Animals and other organisms
☐	☒	Clinical data
☒	☐	Dual use research of concern

Methods

n/a	Involved in the study	
☒	☐	ChIP-seq
☒	☐	Flow cytometry
☒	☐	MRI-based neuroimaging

Antibodies

Antibodies used

Plasma phosphorylated tau 217 (p-tau217) was quantified in both BioFINDER cohorts with an immunoassay developed by Lilly Research Laboratories, and analyses were performed with the same batch of reagents. Biotinylated-IBA493 was used as a capture antibody and SULFO-TAG-4G102 as the detector. In TRIAD, plasma p-tau217 was quantified with an immunoassay developed by Janssen R&D, using the PT3 antibody as capture and HT43 as detector. Samples and antibodies in both assays were diluted in 1:2. These antibodies are research-use only and are not commercialized.

Validation

The plasma p-tau217 immunoassays here used has been previously widely validated for this application and described in full detail (Lilly immunoassay: Palmqvist et al., JAMA. 2020:324(8):772-781; Janssen immunoassay: Groot et al, Alz Res Ther; 2022 May 14;14(1):67).

Clinical data

Policy information about clinical studies

All manuscripts should comply with the ICMJE guidelines for publication of clinical research and a completed CONSORT checklist must be included with all submissions.

Clinical trial registration

BioFINDER-1: NCT01208675. BioFINDER-2: NCT03174938.

Study protocol

BioFINDER-1: https://clinicaltrials.gov/ct2/show/NCT01208675. BioFINDER-2: https://clinicaltrials.gov/ct2/show/NCT03174938.

Data collection

BioFINDER-1 participants were recruited between January 2010 and January 2015. BioFINDER-2 started recruitment in May 2017.

Outcomes

The primary outcome of the study was the overall accuracy of the two-step workflow for Aβ-status, composed of a first step with a blood-biomarker prediction model, and further testing with CSF Aβ42/40 for individuals with uncertain results at the first step. This outcome would inform on how accurate classification was in general, for both steps. Another primary outcome was the number of further confirmatory tests reduced by step-1. Interpreted these two outcomes together enables the evaluation of the trade-off between accuracy and number of confirmatory tests, which is the main focus of the paper. Aβ-PET-status, the reference standard involved in the outcome, was defined by 18-F-flutemetamol PET, using previously validated cutoffs.
